# Supplementary material for: Long-term solid fuel use and risks of major eye diseases in China: A population-based cohort study of 486,532 adults
Source: PLoS Med. 2021 Jul 29;18(7):e1003716. doi: 10.1371/journal.pmed.1003716 (PMC8321372; doi:10.1371/journal.pmed.1003716)
Supplement: S1 STROBE Checklist — (DOCX) [file pmed.1003716.s001.docx]

# STROBE Statement—checklist of items that should be included in reports of observational studies

|  | **Item No.** | **Recommendation** | **Relevant section and paragraph number** |
| --- | --- | --- | --- |
| **Title and abstract** | 1 | (*a*) Indicate the study’s design with a commonly used term in the title or the abstract | Title |
|  |  | (*b*) Provide in the abstract an informative and balanced summary of what was done and what was found | Abstract, paragraph 2-4. |
| Introduction | | | |
| Background/rationale | 2 | Explain the scientific background and rationale for the investigation being reported | Introduction, paragraph 1-2. |
| Objectives | 3 | State specific objectives, including any prespecified hypotheses | Introduction, paragraph 2. |
| Methods | | | |
| Study design | 4 | Present key elements of study design early in the paper | Methods, “Study design” sub-section, paragraph 1-2. |
| Setting | 5 | Describe the setting, locations, and relevant dates, including periods of recruitment, exposure, follow-up, and data collection | Methods, “Study design” sub-section, paragraph 1; Methods, “Follow-up and outcome definition” sub-section, paragraph 1. |
| Participants | 6 | (*a*) *Cohort study*—Give the eligibility criteria, and the sources and methods of selection of participants. Describe methods of follow-up | Methods, “Study design” sub-section, paragraph 1; Methods, “Follow-up and outcome definition” sub-section, paragraph 1. |
|  |  | (*b*) *Cohort study*—For matched studies, give matching criteria and number of exposed and unexposed | Not applicable. |
| Variables | 7 | Clearly define all outcomes, exposures, predictors, potential confounders, and effect modifiers. Give diagnostic criteria, if applicable | Methods, “Assessment of fuel use behaviour and household air pollution exposure” sub-section, paragraph 1-2; Methods, “Follow-up and outcome definition” sub-section, paragraph 1; Methods, “Statistical analysis” sub-section, paragraph 3; Supplementary Appendix, “Supplementary methods” section, paragraph 1-2. |

(Continued on next page)

|  | **Item No.** | **Recommendation** | **Relevant section and paragraph number** |
| --- | --- | --- | --- |
| Data sources/ measurement | 8* | For each variable of interest, give sources of data and details of methods of assessment (measurement). Describe comparability of assessment methods if there is more than one group | Methods, “Assessment of fuel use behaviour and household air pollution exposure” sub-section, paragraph 1-2; Methods, “Follow-up and outcome definition” sub-section, paragraph 1; Methods, “Statistical analysis” sub-section, paragraph 3; Supplementary Appendix, “Supplementary methods” section, paragraph 1-2. |
| Bias | 9 | Describe any efforts to address potential sources of bias | Methods, “Study design” sub-section, paragraph 1; Methods, “Follow-up and outcome definition” sub-section, paragraph 1; Methods, “Statistical analysis” sub-section, paragraph 1. |
| Study size | 10 | Explain how the study size was arrived at | Methods, “Statistical analysis” sub-section, paragraph 1. |
| Quantitative variables | 11 | Explain how quantitative variables were handled in the analyses. If applicable, describe which groupings were chosen and why | Methods, “Assessment of fuel use behaviour and household air pollution exposure” sub-section, paragraph 1-2; Methods, “Statistical analysis” sub-section, paragraph 2. |
| Statistical methods | 12 | (a) Describe all statistical methods, including those used to control for confounding | Methods, “Statistical analysis” sub-section, paragraph 1-3; Supplementary Appendix, “Supplementary methods” section, paragraph 1-2. |
|  |  | (b) Describe any methods used to examine subgroups and interactions | Methods, “Statistical analysis” sub-section, paragraph 3. |
|  |  | (c) Explain how missing data were addressed | Methods, “Statistical analysis” sub-section, paragraph 1. |
|  |  | (d) Cohort study—If applicable, explain how loss to follow-up was addressed | Methods, “Follow-up and outcome definition” sub-section, paragraph 1. |
|  |  | (e) Describe any sensitivity analyses | Methods, “Statistical analysis” sub-section, paragraph 3. |

(Continued on next page)

|  | **Item No.** | **Recommendation** | **Relevant section and paragraph number** |
| --- | --- | --- | --- |
| **Results** |  |  |  |
| Participants | 13* | (a) Report numbers of individuals at each stage of study—eg numbers potentially eligible, examined for eligibility, confirmed eligible, included in the study, completing follow-up, and analysed | Methods, “Statistical analysis” sub-section, paragraph 1. |
|  |  | (b) Give reasons for non-participation at each stage | Methods, “Statistical analysis” sub-section, paragraph 1. |
|  |  | (c) Consider use of a flow diagram | Relevant information was described in text. (Methods, “Statistical analysis” sub-section, paragraph 1) |
| Descriptive data | 14* | (a) Give characteristics of study participants (eg demographic, clinical, social) and information on exposures and potential confounders | Results, paragraph 1. |
|  |  | (b) Indicate number of participants with missing data for each variable of interest | Results, paragraph 1. |
|  |  | (c) *Cohort study*—Summarise follow-up time (eg, average and total amount) | Results, paragraph 2. |
| Outcome data | 15* | *Cohort study*—Report numbers of outcome events or summary measures over time | Results, paragraph 2. |
| Main results | 16 | (*a*) Give unadjusted estimates and, if applicable, confounder-adjusted estimates and their precision (eg, 95% confidence interval). Make clear which confounders were adjusted for and why they were included | Results, paragraph 3-6. |
|  |  | (*b*) Report category boundaries when continuous variables were categorized | Results, paragraph 2-6. |
|  |  | (*c*) If relevant, consider translating estimates of relative risk into absolute risk for a meaningful time period | Odds ratio and hazard ratio were used for effect estimates. |
| Other analyses | 17 | Report other analyses done—e.g. analyses of subgroups and interactions, and sensitivity analyses | Results, paragraph 5-6. |
| **Discussion** | | | |
| Key results | 18 | Summarise key results with reference to study objectives | Discussion, paragraph 1. |

(Continued on next page)

|  | | **Item No.** | **Recommendation** | **Relevant section and paragraph number** |
| --- | --- | --- | --- | --- |
| Limitations | | 19 | Discuss limitations of the study, taking into account sources of potential bias or imprecision. Discuss both direction and magnitude of any potential bias | Discussion, paragraph 9. |
| Interpretation | | 20 | Give a cautious overall interpretation of results considering objectives, limitations, multiplicity of analyses, results from similar studies, and other relevant evidence | Discussion, paragraph 2-8. |
| Generalisability | | 21 | Discuss the generalisability (external validity) of the study results | Discussion, paragraph 8, 10. |
| **Other information** | | | | |
| Funding | 22 | | Give the source of funding and the role of the funders for the present study and, if applicable, for the original study on which the present article is based | Provided as meta-data in the manuscript. |

*Give information separately for cases and controls in case-control studies and, if applicable, for exposed and unexposed groups in cohort and cross-sectional studies.

**Note:** An Explanation and Elaboration article discusses each checklist item and gives methodological background and published examples of transparent reporting. The STROBE checklist is best used in conjunction with this article (freely available on the Web sites of PLoS Medicine at http://www.plosmedicine.org/, Annals of Internal Medicine at http://www.annals.org/, and Epidemiology at http://www.epidem.com/). Information on the STROBE Initiative is available at www.strobe-statement.org.
