# Supplementary material for: Long-term solid fuel use and risks of major eye diseases in China: A population-based cohort study of 486,532 adults
Source: PLoS Med. 2021 Jul 29;18(7):e1003716. doi: 10.1371/journal.pmed.1003716 (PMC8321372; doi:10.1371/journal.pmed.1003716)
Supplement: S1 Text — (DOCX) [file pmed.1003716.s002.docx]

**Supplementary Methods**

**Long-term solid fuel use and risks of major eye diseases in China: a population-based cohort study of 486,532 adults**

Ka Hung Chan^1,2^, Mingshu Yan^3^, Derrick A Bennett^1,4^, Yu Guo^5^, Yiping Chen^1,3^, Ling Yang^1,3^, Jun Lv^6^, Canqing Yu^6^, Pei Pei^5^, Yan Lu^7^, Liming Li^6†^, Huaidong Du^1,3^*, Kin Bong Hubert Lam^1^*, Zhengming Chen^1,3†^ on behalf of the China Kadoorie Biobank Study group

^1^Clinical Trial Service Unit and Epidemiological Studies Unit, Nuffield Department of Population Health, University of Oxford, UK

^2^Oxford British Heart Foundation Centre of Research Excellence, University of Oxford, UK

^3^MRC Population Health Research Unit, Nuffield Department of Population Health, University of Oxford, UK

^4^NIHR Oxford biomedical research Centre, Oxford University Hospitals NHS Foundation Trust, UK

^5^Chinese Academy of Medical Science, Beijing, China

^6^Department of Epidemiology and Biostatistics, School of Public Health, Peking University Health Science Center, Beijing, China

^7^NCD Prevention and Control Department, Suzhou Center for Disease Control and Prevention, Suzhou, China

*Corresponding authors: Dr Huaidong Du, [huaidong.du@ndph.ox.ac.uk](mailto:huaidong.du@ndph.ox.ac.uk) and Dr Kin Bong Hubert Lam, [hubert.lam@ndph.ox.ac.uk](mailto:hubert.lam@ndph.ox.ac.uk)

^†^Senior authors

**Details of the direct standardisation methods**

The original CKB study population was considered as the reference population, structured by 200 strata created from age groups (ten 5-year strata), gender, and ten study areas; then, adjusted percentages or means within each cooking fuel exposure group were estimated as $\frac{\sum_{i} p_{i} \times\omega_{i}}{\sum_{i} \omega_{i}}$, where $p_{i}$ are the raw percentages or means in the i^th^ stratum and $\omega_{i}$ are the weights (i.e. raw number of participant in the same stratum). Adjusted disease incidence rates were computed using the same approach, with adjusted rates estimated as $\frac{\sum_{i} r_{i} \times\omega_{i}}{\sum_{i} \omega_{i}}$, where $r_{i}$are the incidence rates in the i^th^ stratum and $\omega_{i}$ are the person-years at risk for each strata in the reference CKB population) [1].

**Details of covariates adjusted in the primary logistic regression analysis**

Confounders adjusted include age at baseline (continuous), birth cohort (<1940, 1940-1940, 1950-1959, 1960-1969, ≥1970), gender, study area (10 categories), education (no formal education, primary, middle school, ≥high school), occupation (agricultural worker, factory worker, officer worker, home-maker, retiree, self-employed, unemployed or undefined), alcohol intake (current-regular, ex-regular, occasional, never-regular), smoking (current-regular, ex-regular, occasional, never-regular), environmental tobacco smoke (never/ occasional, 1-5 days/ week, 6-7 days/ week), cookstove ventilation (never had ventilation, always some or all ventilated, always all ventilated, mixed), heating fuel exposure (always clean fuels, solid to clean fuels, always solid fuels, never had heating, others), BMI (continuous), prevalent diabetes, self-reported general health, and length of recall period (continuous).

**Details of covariates adjusted in the supplementary Cox regression analysis**

Given the likelihood for age, gender and study areas to violate the proportional hazard assumption, we followed our conventional approach to conduct the Cox regression stratified by age-at-risk (i.e. attained age during the follow-up), gender and study areas in addition to adjustment for education, occupation, alcohol intake, smoking, environmental tobacco smoke, cookstove ventilation, heating fuel exposure, body mass index (BMI), prevalent diabetes, self-reported general health, and length of recall period as in the primary analysis.

**References**

1. Woodward M. Confounding and interaction. Epidemiology: Study Design and Data Analysis. 3rd ed. Oxford, UK: CPC Press; 2014.
