# Supplementary material for: Long-term solid fuel use and risks of major eye diseases in China: A population-based cohort study of 486,532 adults
Source: PLoS Med. 2021 Jul 29;18(7):e1003716. doi: 10.1371/journal.pmed.1003716 (PMC8321372; doi:10.1371/journal.pmed.1003716)
Supplement: S1 Tables — Table A. Major categories of eye disease examined. Table B. Associations between the major eye diseases examined. Table C. Odds ratios and group-specific 95% confidence intervals for major eye diseases according to long-term cooking fuel use—results of sensitivity analysis (1). Table D. Odds ratios and 95% confidence intervals for major eye diseases according to long-term cooking fuel use—results of sensitivity analysis (2). Table E. Odds ratios and 95% confidence intervals for major eye diseases in long-term solid fuel users versus clean fuel users from leave-one-out sensitivity analysis. Table F. Comparison of odds ratios (ORs) of primary analysis and hazard ratios (HRs) estimates from Cox regression analysis. Table G. Comparison of odds ratios (ORs) of primary analysis on duration of solid fuel use and hazard ratios (HRs) estimates from Cox regression analysis. Table H. Comparison of odds ratios (ORs) of primary analysis on types of solid fuel use and hazard ratios (HRs) estimates from Cox regression analysis. Table I. Characteristics of cross-sectional and case–control studies evaluating household air pollution and the risk of cataract. (DOCX) [file pmed.1003716.s003.docx]

**Supplementary Tables**

**Long-term solid fuel use and risks of major eye diseases in China: a population-based cohort study of 486,532 adults**

Ka Hung Chan^1,2^, Mingshu Yan^3^, Derrick A Bennett^1,4^, Yu Guo^5^, Yiping Chen^1,3^, Ling Yang^1,3^, Jun Lv^6^, Canqing Yu^6^, Pei Pei^5^, Yan Lu^7^, Liming Li^6†^, Huaidong Du^1,3^*, Kin Bong Hubert Lam^1^*, Zhengming Chen^1,3†^ on behalf of the China Kadoorie Biobank Study group

^1^Clinical Trial Service Unit and Epidemiological Studies Unit, Nuffield Department of Population Health, University of Oxford, UK

^2^Oxford British Heart Foundation Centre of Research Excellence, University of Oxford, UK

^3^MRC Population Health Research Unit, Nuffield Department of Population Health, University of Oxford, UK

^4^NIHR Oxford biomedical research Centre, Oxford University Hospitals NHS Foundation Trust, UK

^5^Chinese Academy of Medical Science, Beijing, China

^6^Department of Epidemiology and Biostatistics, School of Public Health, Peking University Health Science Center, Beijing, China

^7^NCD Prevention and Control Department, Suzhou Center for Disease Control and Prevention, Suzhou, China

*Corresponding authors: Dr Huaidong Du, [huaidong.du@ndph.ox.ac.uk](mailto:huaidong.du@ndph.ox.ac.uk) and Dr Kin Bong Hubert Lam, [hubert.lam@ndph.ox.ac.uk](mailto:hubert.lam@ndph.ox.ac.uk)

^†^Senior authors

| Table A. Major categories of eye disease examined | | | |
| --- | --- | --- | --- |
|  |  | **Case numbers** | |
| **Disease category according to ICD-10* (specific diseases)** | **ICD-10 code** | **Original cohort** | **In primary analysis** |
| Conjunctiva disorders (conjunctivitis, other disorders of conjunctiva) | H10-H11 | 5,040 | 4,877 |
| Age-related cataracts, non-specific cataracts | H25, H26.9 | 14,142 | 13,396 |
| Disorders of sclera, cornea, iris and ciliary body (disorders of sclera, keratitis, corneal scars and opacities, other disorders of the cornea, iridocyclitis, other disorders of iris and ciliary body, disorders of iris and ciliary body in diseases classified elsewhere) | H15-H22 | 1,647 | 1,583 |
| Glaucoma (glaucoma, glaucoma in diseases classified elsewhere) | H40, H42 | 1,626 | 1,534 |

*ICD10: International Classification of Diseases, 10^th^ revision.

| Table B. Associations between the major eye diseases examined | | | | | | | | |
| --- | --- | --- | --- | --- | --- | --- | --- | --- |
|  |  | **Conjunctiva disorders** | **Cataracts** | | **Disorders of sclera, cornea, iris and ciliary body** | | **Glaucoma** | |
|  | **N** | **OR (95% CI)*** | **n (%)** | **OR (95% CI)*** | **n (%)** | **OR (95% CI)*** | **n (%)** | **OR (95% CI)*** |
| **Conjunctiva disorders** | 4877 | - | 465 (9.5) | 3.46 (3.12-3.84) | 246 (5.0) | 7.11 (6.14-8.22) | 56 (1.2) | 3.71 (2.81-4.90) |
| **Cataracts** | 13,396 | - | - | - | 180 (1.3) | 4.15 (3.52-4.90) | 295 (2.2) | 5.09 (4.44-5.83) |
| **Disorders of sclera, cornea, iris and ciliary body** | 1534 | - | - | - | - | - | 51 (3.2) | 10.3 (7.68-13.8) |
| **Glaucoma** | 1583 | - | - | - | - | - | - | - |
| *Odds ratios were adjusted for age, gender, birth cohort, education and occupation. | | | |  |  |  |  |  |

| **Table C. Odds ratios and group-specific 95% confidence intervals for major eye diseases according to long-term cooking fuel use - results of sensitivity analysis (1)** | | | | | | | | | | |  |
| --- | --- | --- | --- | --- | --- | --- | --- | --- | --- | --- | --- |
|  | **Primary analysis (n = 486,532)*** | | **Additional adjustment (n = 486,532)†** | | **Excluding weekly cook (n = 426,741)** | | **Excluding frequent movers (n = 455,860)** | | **Excluding prevalent diabetes (n = 458,234) ^‡^** | | |
|  | **No. of events** | **OR (95% CI)** | **No. of events** | **OR (95% CI)** | **No. of events** | **OR (95% CI)** | **No. of events** | **OR (95% CI)** | **No. of events** | **OR (95% CI)** | |
| **Conjunctiva disorders** |  |  |  |  |  |  |  |  |  |  | |
| Always clean (reference) | 465 | 1.00 (0.91-1.10) | 465 | 1.00 (0.90-1.11) | 332 | 1.00 (0.89-1.12) | 407 | 1.00 (0.90-1.11) | 433 | 1.00 (0.90-1.11) | |
| Solid to clean | 758 | 1.21 (1.12-1.31) | 758 | 1.21 (1.11-1.31) | 632 | 1.14 (1.04-1.24) | 707 | 1.21 (1.12-1.32) | 708 | 1.22 (1.12-1.33) | |
| Always solid | 2350 | 1.32 (1.25-1.39) | 2350 | 1.33 (1.26-1.41) | 2018 | 1.24 (1.17-1.32) | 2286 | 1.34 (1.27-1.42) | 2249 | 1.33 (1.25-1.40) | |
| **Cataracts** |  |  |  |  |  |  |  |  |  |  | |
| Always clean (reference) | 1802 | 1.00 (0.95-1.06) | 1802 | 1.00 (0.95-1.06) | 1415 | 1.00 (0.94-1.06) | 1594 | 1.00 (0.94-1.06) | 1463 | 1.00 (0.94-1.06) | |
| Solid to clean | 3141 | 1.05 (1.00-1.10) | 3141 | 1.04 (0.99-1.09) | 2718 | 1.05 (1.00-1.10) | 3015 | 1.04 (0.99-1.09) | 2548 | 1.06 (1.00-1.11) | |
| Always solid | 5400 | 1.17 (1.11-1.22) | 5400 | 1.13 (1.08-1.18) | 4845 | 1.18 (1.12-1.23) | 5251 | 1.16 (1.11-1.22) | 4910 | 1.17 (1.11-1.22) | |
| **Disorders of sclera, cornea, iris and ciliary body** |  |  |  |  |  |  |  |  |  |  | |
| Always clean (reference) | 149 | 1.00 (0.84-1.19) | 149 | 1.00 (0.84-1.19) | 97 | 1.00 (0.81-1.24) | 127 | 1.00 (0.83-1.20) | 139 | 1.00 (0.84-1.20) | |
| Solid to clean | 232 | 1.21 (1.04-1.40) | 232 | 1.21 (1.04-1.41) | 187 | 1.20 (1.02-1.41) | 214 | 1.17 (1.00-1.37) | 204 | 1.17 (1.00-1.37) | |
| Always solid | 721 | 1.35 (1.22-1.48) | 721 | 1.31 (1.18-1.45) | 632 | 1.41 (1.27-1.57) | 695 | 1.33 (1.20-1.46) | 681 | 1.33 (1.20-1.47) | |
| **Glaucoma** |  |  |  |  |  |  |  |  |  |  | |
| Always clean (reference) | 253 | 1.00 (0.87-1.15) | 253 | 1.00 (0.87-1.15) | 207 | 1.00 (0.86-1.17) | 220 | 1.00 (0.86-1.16) | 220 | 1.00 (0.86-1.17) | |
| Solid to clean | 449 | 1.00 (0.89-1.13) | 449 | 1.01 (0.89-1.14) | 387 | 0.96 (0.85-1.09) | 426 | 0.92 (0.82-1.05) | 381 | 0.99 (0.87-1.13) | |
| Always solid | 520 | 0.95 (0.82-1.09) | 520 | 0.94 (0.81-1.09) | 484 | 0.97 (0.83-1.13) | 499 | 0.89 (0.76-1.03) | 477 | 0.93 (0.80-1.08) | |

* Odds ratios were adjusted for age at baseline, birth cohort, gender, study area, education, occupation, smoking, environmental tobacco smoke, cookstove ventilation, heating fuel exposure, BMI, prevalent diabetes, self-reported general health, and length of recall period.
^†^ Odds ratios were further adjusted for diet (meat, fresh fruit, dairy products, eggs), physical activity level, and random blood glucose level at baseline.
**^‡^** Prevalent diabetes refers to self-reported history of physician-diagnosis of diabetes or screen-detected diabetes based on random or fasting blood glucose levels at baseline.

| **Table D. Odds ratios and 95% confidence intervals for major eye diseases according to long-term cooking fuel use - results of sensitivity analysis (2)** | | | | | | | | | | |
| --- | --- | --- | --- | --- | --- | --- | --- | --- | --- | --- |
|  | **Excluding poor self-rated health (n = 437,052)** | | **Excluding those aged ≥65 years (n = 411,846)** | | **Excluding those aged <40 years (n = 410,102)** | | **Excluding first 3 years follow-up^†^** | | **Excluding cases diagnosed within 1 year after the diagnosis of another eye disease^‡^** | |
|  | **No. of events** | **OR (95% CI)** | **No. of events** | **OR (95% CI)** | **No. of events** | **OR (95% CI)** | **No. of events** | **OR (95% CI)** | **No. of events** | **OR (95% CI)** |
| **Conjunctiva disorders** |  |  |  |  |  |  |  |  |  |  |
| Always clean (reference) | 436 | 1.00 (0.90-1.11) | 414 | 1.00 (0.90-1.11) | 410 | 1.00 (0.90-1.11) | 248 | 1.00 (0.87-1.15) | 442 | 1.00 (0.90-1.11) |
| Solid to clean | 701 | 1.23 (1.13-1.34) | 652 | 1.22 (1.12-1.33) | 727 | 1.21 (1.11-1.31) | 465 | 1.36 (1.21-1.52) | 730 | 1.24 (1.14-1.35) |
| Always solid | 2097 | 1.28 (1.21-1.35) | 2014 | 1.30 (1.23-1.39) | 2194 | 1.33 (1.26-1.41) | 1548 | 1.63 (1.51-1.76) | 2249 | 1.35 (1.27-1.42) |
| **Cataracts** |  |  |  |  |  |  |  |  |  |  |
| Always clean (reference) | 1650 | 1.00 (0.94-1.06) | 1016 | 1.00 (0.93-1.08) | 1769 | 1.00 (0.95-1.06) | 1528 | 1.00 (0.94-1.06) | 1765 | 1.00 (0.95-1.06) |
| Solid to clean | 2756 | 1.04 (0.99-1.09) | 1529 | 1.08 (1.01-1.15) | 3133 | 1.04 (0.99-1.09) | 2695 | 1.03 (0.98-1.09) | 3071 | 1.05 (1.00-1.10) |
| Always solid | 4515 | 1.16 (1.11-1.22) | 3311 | 1.24 (1.17-1.31) | 5293 | 1.15 (1.10-1.21) | 4827 | 1.12 (1.07-1.18) | 5251 | 1.16 (1.11-1.22) |
| **Disorders of sclera, cornea, iris and ciliary body** |  |  |  |  |  |  |  |  |  |  |
| Always clean (reference) | 143 | 1.00 (0.84-1.19) | 123 | 1.00 (0.83-1.21) | 133 | 1.00 (0.83-1.20) | 83 | 1.00 (0.78-1.29) | 125 | 1.00 (0.83-1.21) |
| Solid to clean | 206 | 1.17 (1.00-1.37) | 189 | 1.29 (1.10-1.51) | 222 | 1.16 (1.00-1.35) | 132 | 1.14 (0.92-1.42) | 207 | 1.34 (1.14-1.56) |
| Always solid | 664 | 1.31 (1.18-1.45) | 619 | 1.41 (1.26-1.56) | 670 | 1.31 (1.18-1.45) | 444 | 1.31 (1.14-1.51) | 644 | 1.43 (1.29-1.58) |
| **Glaucoma** |  |  |  |  |  |  |  |  |  |  |
| Always clean (reference) | 231 | 1.00 (0.86-1.16) | 164 | 1.00 (0.84-1.20) | 246 | 1.00 (0.87-1.15) | 195 | 1.00 (0.85-1.18) | 242 | 1.00 (0.87-1.15) |
| Solid to clean | 404 | 1.01 (0.89-1.15) | 287 | 1.16 (1.00-1.35) | 447 | 0.99 (0.88-1.11) | 330 | 0.97 (0.84-1.12) | 425 | 1.00 (0.89-1.13) |
| Always solid | 456 | 0.89 (0.77-1.04) | 393 | 1.06 (0.89-1.27) | 498 | 0.93 (0.80-1.07) | 402 | 0.92 (0.77-1.09) | 478 | 0.98 (0.84-1.14) |

* Odds ratios were adjusted for age at baseline, birth cohort, gender, study area, education, occupation, smoking, environmental tobacco smoke, cookstove ventilation, heating fuel exposure, BMI, prevalent diabetes, self-reported general health, and length of recall period.

^†^ Excluded 1843 of 4877 conjunctiva disorders, 1681 of 13,396 cataracts, 656 of 1583 disorders of sclera, cornea, iris and ciliary body, and 17 of 1534 glaucoma cases.

^‡^ Excluded 204 of 4877 conjunctiva disorders, 317 of 13,396 cataracts, 175 of 1583 disorders of sclera, cornea, iris and ciliary body, and 102 of 1534 glaucoma cases.

| Table E. Odds ratios and 95% confidence intervals for major eye diseases in long-term solid fuel users versus clean fuel users from leave-one-out sensitivity analysis | | | | | | | | | | | |
| --- | --- | --- | --- | --- | --- | --- | --- | --- | --- | --- | --- |
|  | **Disorders of conjunctiva** | |  | **Cataracts** | |  | **Disorders of sclera, cornea, iris and ciliary body** | |  | **Glaucoma** | |
|  | **Number of**  **cases** | **OR (95% CI)** |  | **Number of  cases** | **OR (95% CI)** |  | **Number of cases** | **OR (95% CI)** |  | **Number of  cases** | **OR (95% CI)** |
| **Qingdao excluded** | 4740 | 1.32 (1.17-1.49) |  | 11,861 | 1.15 (1.06-1.25) |  | 1572 | 1.33 (1.08-1.64) |  | 1433 | 0.97 (0.77-1.22) |
| **Harbin excluded** | 4817 | 1.33 (1.18-1.49) |  | 11,677 | 1.13 (1.04-1.22) |  | 1532 | 1.42 (1.15-1.77) |  | 1185 | 1.01 (0.79-1.30) |
| **Haikou excluded** | 4574 | 1.16 (1.03-1.31) |  | 12,591 | 1.14 (1.05-1.24) |  | 1563 | 1.31 (1.06-1.61) |  | 1470 | 0.96 (0.76-1.20) |
| **Suzhou excluded** | 4663 | 1.32 (1.17-1.49) |  | 12,912 | 1.17 (1.08-1.26) |  | 1564 | 1.35 (1.09-1.66) |  | 1436 | 0.98 (0.78-1.23) |
| **Liuzhou excluded** | 4698 | 1.32 (1.17-1.49) |  | 12,145 | 1.19 (1.09-1.29) |  | 1469 | 1.32 (1.06-1.64) |  | 1340 | 0.90 (0.71-1.14) |
| **Sichuan excluded** | 4678 | 1.35 (1.20-1.52) |  | 11,710 | 1.17 (1.08-1.26) |  | 1536 | 1.33 (1.08-1.64) |  | 1470 | 0.97 (0.77-1.21) |
| **Gansu excluded** | 4591 | 1.31 (1.16-1.47) |  | 12,540 | 1.16 (1.07-1.25) |  | 1521 | 1.35 (1.10-1.66) |  | 1471 | 0.92 (0.74-1.15) |
| **Henan excluded** | 4723 | 1.32 (1.17-1.49) |  | 12,192 | 1.16 (1.08-1.25) |  | 1545 | 1.36 (1.10-1.67) |  | 1337 | 0.89 (0.71-1.11) |
| **Zhejiang excluded** | 2465 | 1.76 (1.45-2.14) |  | 11,846 | 1.22 (1.12-1.32) |  | 777 | 1.55 (1.06-2.25) |  | 1377 | 0.90 (0.70-1.14) |
| **Hunan excluded** | 3944 | 1.29 (1.14-1.45) |  | 11,090 | 1.18 (1.09-1.27) |  | 1168 | 1.33 (1.07-1.65) |  | 1287 | 0.99 (0.79-1.25) |

* Odds ratios were adjusted for age at baseline, birth cohort, gender, study area, education, occupation, smoking, environmental tobacco smoke, cookstove ventilation, heating fuel exposure, BMI, prevalent diabetes, self-reported general health, and length of recall period. Conventional 95% confidence intervals are presented with reference to long-term clean fuel users.

| Table F. Comparison of odds ratios (ORs) of primary analysis and hazard ratios (HRs) estimates from Cox regression analysis | | | | |
| --- | --- | --- | --- | --- |
|  | **Logistic regression analysis** | |  | **Cox regression analysis** |
|  | **Event no.** | **OR (95% CI)*** |  | **HR (95% CI)^†^** |
| **Disorders of conjunctiva** |  |  |  |  |
| Always clean (reference) | 465 | 1.00 (0.91-1.10) |  | 1.00 (0.91-1.10) |
| Solid to clean | 758 | 1.22 (1.13-1.32) |  | 1.14 (1.05-1.23) |
| Always solid | 2350 | 1.33 (1.26-1.40) |  | 1.26 (1.19-1.33) |
| Never-regular cook^‡^ | 1304 | 1.29 (1.20-1.39) |  | 1.20 (1.12-1.30) |
| **Cataracts** |  |  |  |  |
| Always clean (reference) | 1802 | 1.00 (0.95-1.06) |  | 1.00 (0.95-1.05) |
| Solid to clean | 3141 | 1.05 (1.00-1.10) |  | 1.00 (0.95-1.05) |
| Always solid | 5400 | 1.17 (1.11-1.22) |  | 1.06 (1.01-1.11) |
| Never-regular cook^‡^ | 3053 | 1.08 (1.03-1.14) |  | 1.08 (1.03-1.14) |
| **Disorders of sclera, cornea, iris and ciliary body** |  |  |  |  |
| Always clean (reference) | 149 | 1.00 (0.84-1.19) |  | 1.00 (0.84-1.19) |
| Solid to clean | 232 | 1.22 (1.06-1.42) |  | 1.11 (0.95-1.29) |
| Always solid | 721 | 1.37 (1.25-1.51) |  | 1.30 (1.17-1.44) |
| Never-regular cook^‡^ | 481 | 1.29 (1.15-1.45) |  | 1.22 (1.09-1.37) |
| **Glaucoma** |  |  |  |  |
| Always clean (reference) | 253 | 1.00 (0.87-1.15) |  | 1.00 (0.87-1.15) |
| Solid to clean | 449 | 1.00 (0.89-1.13) |  | 0.95 (0.84-1.07) |
| Always solid | 520 | 0.95 (0.82-1.10) |  | 0.85 (0.71-1.00) |
| Never-regular cook^‡^ | 312 | 1.08 (0.93-1.25) |  | 1.09 (0.93-1.27) |

*Adjusted for baseline age, birth cohort, gender, study area, education, occupation, smoking, passive smoking, cookstove ventilation, heating fuel exposure, prevalent diabetes, body mass index, length of recall period, and self-reported general health.
†Stratified by age-at-risk, gender, and study area, and adjusted for education, occupation, smoking, passive smoking, cookstove ventilation, heating fuel exposure, prevalent diabetes, body mass index, length of recall period, and self-reported general health.
^‡^ Never-regular cook: individuals who reported cooking for monthly or less frequently throughout the recall period.

| Table G. Comparison of odds ratios (ORs) of primary analysis on duration of solid fuel use and hazard ratios (HRs) estimates from Cox regression analysis | | | | |
| --- | --- | --- | --- | --- |
|  | **Logistic regression analysis** | |  | **Cox regression analysis** |
|  | **Event no.** | **OR (95% CI)*** |  | **HR (95% CI)^†^** |
| **Disorders of conjunctiva** |  |  |  |  |
| Always clean (reference) | 465 | 1.00 (0.90-1.11) |  | 1.00 (0.90-1.11) |
| Solid to clean | 758 | 1.23 (1.13-1.33) |  | 1.13 (1.04-1.23) |
| Always solid <20 years | 544 | 1.29 (1.20-1.38) |  | 1.21 (1.11-1.32) |
| Always solid 20-39 years | 1211 | 1.38 (1.29-1.48) |  | 1.24 (1.16-1.33) |
| Always solid ≥40 years | 595 | 1.34 (1.20-1.49) |  | 1.24 (1.17-1.31) |
| **Cataracts** |  |  |  |  |
| Always clean (reference) | 1802 | 1.00 (0.94-1.06) |  | 1.00 (0.94-1.06) |
| Solid to clean | 3141 | 1.05 (1.00-1.10) |  | 1.00 (0.95-1.05) |
| Always solid <20 years | 754 | 1.09 (1.01-1.18) |  | 1.04 (0.96-1.12) |
| Always solid 20-39 years | 2246 | 1.21 (1.15-1.27) |  | 1.09 (1.04-1.15) |
| Always solid ≥40 years | 2400 | 1.16 (1.10-1.22) |  | 1.08 (1.04-1.11) |
| **Disorders of sclera, cornea, iris and ciliary body** |  |  |  |  |
| Always clean (reference) | 149 | 1.00 (0.84-1.20) |  | 1.00 (0.84-1.19) |
| Solid to clean | 232 | 1.22 (1.05-1.42) |  | 1.09 (0.94-1.28) |
| Always solid <20 years | 193 | 1.35 (1.20-1.52) |  | 1.24 (1.07-1.44) |
| Always solid 20-39 years | 362 | 1.45 (1.28-1.65) |  | 1.24 (1.10-1.41) |
| Always solid ≥40 years | 166 | 1.21 (0.97-1.50) |  | 1.26 (1.14-1.38) |
| **Glaucoma** |  |  |  |  |
| Always clean (reference) | 253 | 1.00 (0.86-1.17) |  | 1.00 (0.86-1.17) |
| Solid to clean | 449 | 1.01 (0.88-1.15) |  | 0.96 (0.83-1.10) |
| Always solid <20 years | 87 | 0.91 (0.76-1.10) |  | 0.87 (0.68-1.10) |
| Always solid 20-39 years | 246 | 0.92 (0.79-1.08) |  | 0.82 (0.69-0.97) |
| Always solid ≥40 years | 187 | 1.07 (0.88-1.29) |  | 1.03 (0.92-1.15) |

*Adjusted for baseline age, birth cohort, gender, study area, education, occupation, smoking, passive smoking, cookstove ventilation, heating fuel exposure, prevalent diabetes, body mass index, and self-reported general health.
†Stratified by age-at-risk, gender, and study area, and adjusted for education, occupation, smoking, passive smoking, cookstove ventilation, heating fuel exposure, prevalent diabetes, body mass index, and self-reported general health.

| Table H. Comparison of odds ratios (ORs) of primary analysis on types of solid fuel use and hazard ratios (HRs) estimates from Cox regression analysis | | | | |
| --- | --- | --- | --- | --- |
|  | **Logistic regression analysis** | |  | **Cox regression analysis** |
|  | **Event no.** | **OR (95% CI)*** |  | **HR (95% CI)^†^** |
| **Disorders of conjunctiva** |  |  |  |  |
| Always clean (reference) | 465 | 1.00 (0.90-1.11) |  | 1.00 (0.90-1.11) |
| Always coal | 477 | 1.32 (1.17-1.49) |  | 1.18 (1.03-1.35) |
| Mix of coal and wood | 336 | 1.38 (1.22-1.57) |  | 1.25 (1.09-1.44) |
| Always wood | 1537 | 1.33 (1.25-1.41) |  | 1.27 (1.19-1.35) |
| **Cataracts** |  |  |  |  |
| Always clean (reference) | 1802 | 1.00 (0.94-1.06) |  | 1.00 (0.94-1.06) |
| Always coal | 1716 | 1.17 (1.09-1.24) |  | 1.01 (0.94-1.08) |
| Mix of coal and wood | 1208 | 1.19 (1.11-1.28) |  | 1.08 (1.00-1.16) |
| Always wood | 2476 | 1.16 (1.10-1.22) |  | 1.08 (1.03-1.14) |
| **Disorders of sclera, cornea, iris and ciliary body** |  |  |  |  |
| Always clean (reference) | 253 | 1.00 (0.84-1.19) |  | 1.00 (0.84-1.19) |
| Always coal | 239 | 1.26 (1.04-1.52) |  | 1.17 (0.95-1.45) |
| Mix of coal and wood | 95 | 1.30 (1.04-1.62) |  | 1.30 (1.02-1.66) |
| Always wood | 186 | 1.41 (1.26-1.58) |  | 1.33 (1.18-1.49) |
| **Glaucoma** |  |  |  |  |
| Always clean (reference) | 149 | 1.00 (0.86-1.16) |  | 1.00 (0.86-1.16) |
| Always coal | 174 | 1.01 (0.83-1.21) |  | 0.80 (0.64-1.00) |
| Mix of coal and wood | 109 | 0.89 (0.70-1.13) |  | 0.84 (0.65-1.09) |
| Always wood | 438 | 0.93 (0.78-1.11) |  | 0.87 (0.73-1.06) |

*Adjusted for baseline age, birth cohort, gender, study area, education, occupation, smoking, passive smoking, cookstove ventilation, heating fuel exposure, prevalent diabetes, body mass index, length of recall period, and self-reported general health.
†Stratified by age-at-risk, gender, and study area, and adjusted for education, occupation, smoking, passive smoking, cookstove ventilation, heating fuel exposure, prevalent diabetes, body mass index, length of recall period, and self-reported general health.

| **Table I. Characteristics of cross-sectional and case-control studies evaluating household air pollution and the risk of cataract** | | | | | | |
| --- | --- | --- | --- | --- | --- | --- |
| Author, year (location) | **Population, (sample size [case number], age[year])** | **Measurement of exposure** | **Outcome** | | | **Confounding variables**  **adjusted** |
|  |  |  | **Case definition**  **of cataract** | **Measurement**  **of cataract** | **Type(s) of cataract** |  |
| Cross-sectional studies | | | | | | |
| Ravilla TD, 2016 (India)[2] | Residents in the catchment area of two eye hospitals, 5871 (4098), ≥ 60 | Self-reported types of cooking fuels and stoves, and length of fuels and stoves use. | Lens Opacities Classification System III (LOCS III) | slit-lamp photographs | nuclear, cortical or posterior-subcapsular cataract | age, gender, region, socioeconomic status, sun exposure, smoking, kitchen type, nutrition, vitamin C deficiency, diabetes. |
| Pokhrel AK, 2013 (Nepal)[3] | Hospital female outpatients, 143 (80), 20-65 | Self-reported type of cooking and heating fuel, and ventilation status in kitchen. | LOCS III | slit-lamp photographs | nuclear or cortical cataract | age, gender, education, smoking, occupation, income, nutritional status, region (diabetes patients were excluded) |
| Saha A, 2005 (India)[4] | Villagers, 469 (around 150), N.A. (mean age 36) | Self-reported household cooking fuel type | N.A. | ophthalmoscopy and slit lamp examination | age-related cataract | age, gender, income, smoking, diabetes, hypertension, house type |
| Case-control studies | | | | | | |
| Das GK, 2019 (India)[5] | hospital outpatients, 90 cases and 90 controls, 18-40 | Self-reported daily outdoor and/or indoor fuel exposure amount | Visual symptoms due to cataract and/or diagnosed as having cataract based on slit lamp examination | slit lamp examination | presenile cataract | only crude analysis was conducted |
| Tanchangya J, 2011 (Bangladesh)[6] | Hospital patients, 153 cases and 306 controls, 18-49 | Self-reported type and length of household cooking fuel use, and cooking frequency. | Clinical diagnose of cataract and confirmed by slit-lamp examination, or having a pseudophakic lens as a result of cataract surgery within the previous 5 years | patient eye examination record | presenile cataract | age, gender, education, family history of cataract, smoking (study was matched on age and gender; diabetes patients were excluded) |
| (cont.) | | | | | | |

| Table I. Characteristics of cross-sectional and case-control studies evaluating household air pollution and the risk of cataract (continued) | | | | | | |
| --- | --- | --- | --- | --- | --- | --- |
| Author, year (location) | **Population, (sample size [case number], age[year])** | **Measurement of exposure** | **Outcome** | | | **Confounding variables**  **adjusted** |
|  |  |  | **Case definition**  **of cataract** | **Measurement**  **of cataract** | **Type(s) of cataract** |  |
| Pokhrel AK, 2005 (Nepal)[7] | Hospital female patients, 206 cases and 203 controls, 35-75 | Self-reported type and length of cooking fuel use, and ventilation status in kitchen. | N.A. | slit lamp examination | age-related cataract | literacy, residency, occupation, ventilation in kitchen, source of light, vegetable intake, milk intake, whether work outside, house type, age stared cooking (study was matched on age; diabetes patients were excluded) |
| Sreenivas V, 1999 (India)[8] | Villagers, 559 cases and 899 controls, 40-60 | Self-reported household cooking fuel type. | On oblique illumination, a lens showing greyness or spokes of grey opacity with or without the presence of iris shadow. | uniocular loupe and ophthalmoscopy examination | age-related cataract | age, gender, alcohol, systolic blood pressure, diastolic blood pressure, pulse rate, height, vitamin supplement, daily sunlight exposure length, exposure to fire/dust, daily work hour, chewing habit. |
| Zodpey, 1999 (India)[9] | Hospital female patients, 223 cases and 223 controls, 35-75 | Self-reported household cooking fuel type. | With a lens opacity sufficient to reduce corrected visual acuity to 6/60 or worse in the affected eye | patient eye examination record | age-related cataract | socio-economic status |
| Ughade SN, 1998 (India)[10] | Ophthalmology clinic patients, 262 cases and 262 controls, 50-80 | Self-reported household cooking fuel type. | N.A. | patient eye examination record | age-related cataract | socio-economic status, education, history of diarrhoea, history of diabetes, glaucoma, myopia early in life, smoking, hypertension (study was matched on age and gender) |
| Mohan M, 1989 (India)[11] | Ophthalmology clinic outpatients, 1441 cases and 549 controls, 37-62 | Self-reported household cooking fuel type. | N.A. | patient's eye examination record | age-related cataract | age, gender, education, dietary protein, BMI, systolic blood pressure, aspirin use (diabetes patients were excluded) |

N.A.: not available – where the relevant information is not reported
