# Supplementary material for: Long-term solid fuel use and risks of major eye diseases in China: A population-based cohort study of 486,532 adults
Source: PLoS Med. 2021 Jul 29;18(7):e1003716. doi: 10.1371/journal.pmed.1003716 (PMC8321372; doi:10.1371/journal.pmed.1003716)
Supplement: S1 Figs — Fig A. Graphical illustration of potential bias from the disproportionately delayed treatment or diagnosis in solid fuel users. Fig B. Associations of cookstove ventilation availability with major eye disease incidence in long-term solid fuel users (DOCX) [file pmed.1003716.s004.docx]

**Supplementary Figures**

**Long-term solid fuel use and risks of major eye diseases in China: a population-based cohort study of 486,532 adults**

Ka Hung Chan^1,2^, Mingshu Yan^3^, Derrick A Bennett^1,4^, Yu Guo^5^, Yiping Chen^1,3^, Ling Yang^1,3^, Jun Lv^6^, Canqing Yu^6^, Pei Pei^5^, Yan Lu^7^, Liming Li^6†^, Huaidong Du^1,3^*, Kin Bong Hubert Lam^1^*, Zhengming Chen^1,3†^ on behalf of the China Kadoorie Biobank Study group

^1^Clinical Trial Service Unit and Epidemiological Studies Unit, Nuffield Department of Population Health, University of Oxford, UK

^2^Oxford British Heart Foundation Centre of Research Excellence, University of Oxford, UK

^3^MRC Population Health Research Unit, Nuffield Department of Population Health, University of Oxford, UK

^4^NIHR Oxford biomedical research Centre, Oxford University Hospitals NHS Foundation Trust, UK

^5^Chinese Academy of Medical Science, Beijing, China

^6^Department of Epidemiology and Biostatistics, School of Public Health, Peking University Health Science Center, Beijing, China

^7^NCD Prevention and Control Department, Suzhou Center for Disease Control and Prevention, Suzhou, China

*Corresponding authors: Dr Huaidong Du, [huaidong.du@ndph.ox.ac.uk](mailto:huaidong.du@ndph.ox.ac.uk) and Dr Kin Bong Hubert Lam, [hubert.lam@ndph.ox.ac.uk](mailto:hubert.lam@ndph.ox.ac.uk)

^†^Senior authors

# Fig A. Graphical illustration of potential bias from the disproportionately delayed treatment or diagnosis in solid fuel users

Baseline

Diagnosis

Detectable cataract

Diagnosis

**Clean fuel users tend to have higher SES and live in urban areas**

**Solid fuel users tend to have lower SES and live in rural areas**

**Observed time-to-event (1)**

**Observed time-to-event (2)**

**Actual time-to-event**

In this hypothetical scenario, assuming clean fuel and solid fuel users developed clinically detectable cataract at the same rate (i.e. no differences in risk as per the null hypothesis), since clean fuel users tend to have higher socioeconomic status (SES) and live in urban areas compared with solid fuel users, they have a higher chance of getting a timely diagnosis or treatment for their eye diseases such as cataracts because of better access to healthcare.[1] The disproportionately longer delays in solid fuel users would generate misleading results suggesting that solid fuel users have a lower rate (due to longer event-free period) of cataracts than clean fuel users in conventional survival analysis. In contrast, logistic regression only examines event counts and is not affected by the timing of the events.

# Fig B. Associations of cookstove ventilation availability with major eye disease incidence in long-term solid fuel users


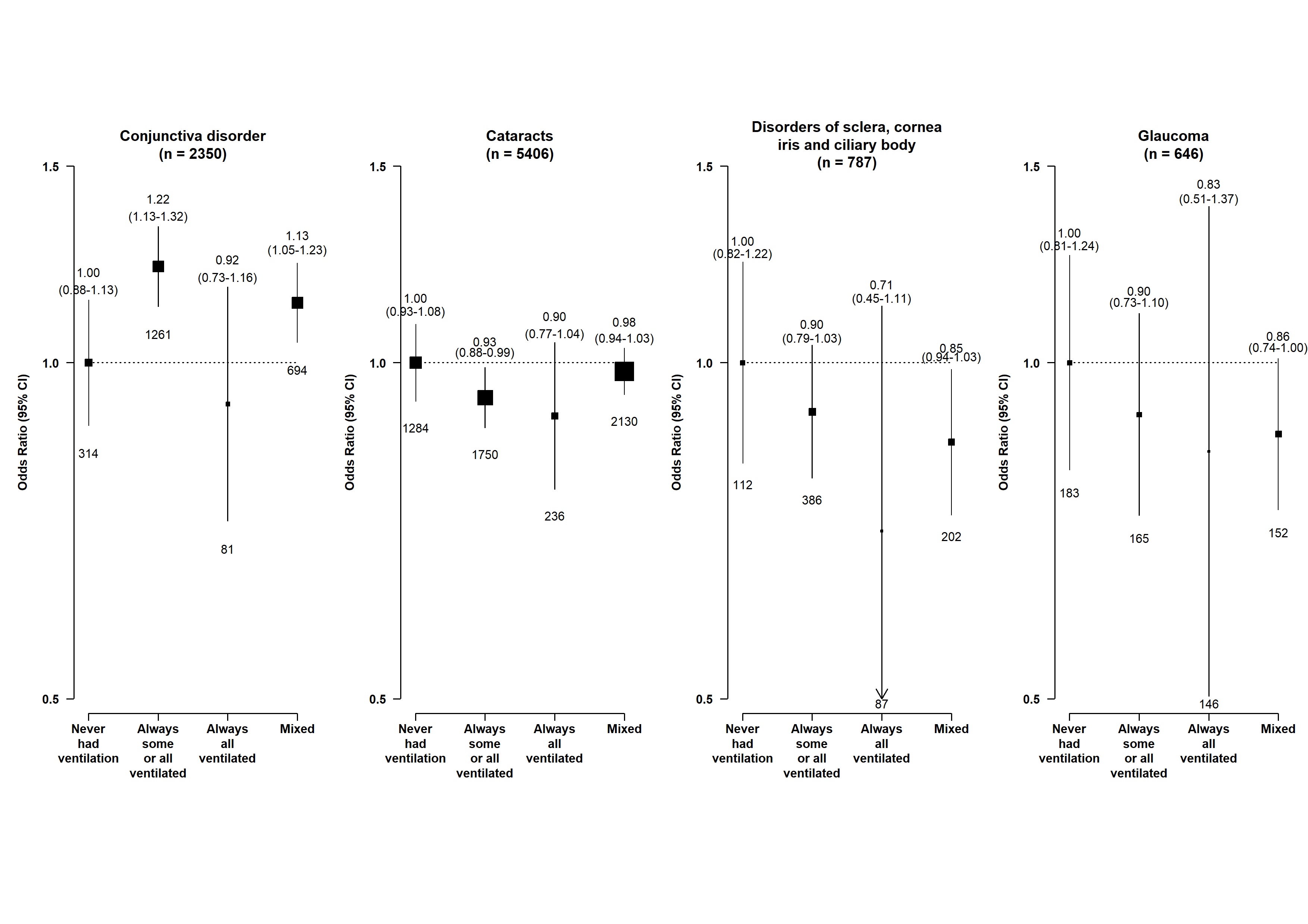


*Odds ratios (ORs) were adjusted for age at baseline, birth cohort, study area, education, occupation, smoking, environmental tobacco smoke, heating fuel exposure, BMI, prevalent diabetes, self-reported general health, and length of recall period. The numbers in brackets are the total case number included in the four comparison groups for each disease endpoint. The boxes represent ORs, with the size inversely proportional to the variance of the logarithm of the category-specific log risk [which also determines the confidence intervals (CIs) represented by the vertical lines]. The numbers above the vertical lines are point estimates for ORs, and the numbers below the lines are numbers of events. Analyses were restricted to long-term solid fuel users (n = 173,288).

# References

1. World Health Organisation. World report on vision. Switzerland: World Health Organization; 2019.
