# Supplementary material for: Long-term solid fuel use and risks of major eye diseases in China: A population-based cohort study of 486,532 adults
Source: PLoS Med. 2021 Jul 29;18(7):e1003716. doi: 10.1371/journal.pmed.1003716 (PMC8321372; doi:10.1371/journal.pmed.1003716)
Supplement: Attachment — Submitted filename: PMEDICINE-D-20-04664R1_Response_27Nov2020.docx [file pmed.1003716.s005.docx]

27 November 2020

Richard Turner, PhD
Senior Editor
PLOS Medicine

Dear Dr Turner,

**Re: PMEDICINE-D-20-04664R1**

Thank you for the opportunity to revise the above manuscript for further consideration for publication in the PLOS Medicine. We have revised the manuscript in the light of the helpful feedback from the reviewers. Attached are our point-by-point responses to the comments. Verbatim comments are shown below in bold, and our responses are shown in non-bold text, with page and line numbers indicating changes in the revised manuscript.

Huaidong Du
Senior Research Fellow

Nuffield Department of Population Health
University of Oxford
United Kingdom

Kin Bong Hubert Lam
Associate Professor

Nuffield Department of Population Health
University of Oxford
United Kingdom

**Response to comments**

Manuscript no.: PMEDICINE-D-20-04664R1

***Editors:***

**C1: We think that you are reporting a retrospective analysis of a prospectively-gathered dataset. Therefore, please remove the word "prospective" from the title, and adapt mentions of this word throughout the text to refer to the parent study rather than the current analysis.**

Response: We appreciate the editors’ perspective, but we believe that since the exposure data were collected prior to disease development and the participants were prospectively followed up for incident disease events, it would qualify the present study as a prospective cohort design. Further details about the China Kadoorie Biobank (CKB) have been published previously [1] and can be found on <https://www.ckbiobank.org>. More in-depth discussion about the prospective nature of the present study is presented in our response to Reviewer 4 C1.

**C2: We suggest moving "in China" before the colon in your title, with the study descriptor then becoming "a population-based cohort study", as noted elsewhere in your paper.**

Response: Done.

**C3: In your abstract and throughout the text, please quote p values alongside 95% CI, where available.**

Response: We have added p values alongside 95% CI in the abstract and results section of the revised manuscript.

**C4: Please make that "A questionnaire ..." in your abstract.**

Response: Done.

**C5: Please add a new final sentence to the "Methods and findings" subsection of your abstract, quoting 2-3 of the study's main limitations.**

Response: Done.

**C6: After the abstract, we will need to ask you to add a new and accessible "Author summary" section in non-identical prose. You may find it helpful to take a look at one or two recent research papers in PLOS Medicine to get a sense of the preferred style.**

Response: We have added an “Author summary” section in the revised manuscript as suggested (see pages 3-4).

**C7: Please remove the information on funding from the title page, the "Role of the funding source" statement from the end of the methods section, and the information on funding and competing interests from the end of the main text. In the event of publication, this information will appear in the article metadata, via entries in the submission form.**

Response: The information has been removed as requested.

**C8: Early in the methods section of your main text, please state whether the present analysis had a protocol or prespecified analysis plan, and if so attach the relevant document(s) as a supplementary file(s) in the text. Please highlight analyses that were not prespecified, including any carried out in response to referees' comments.**

Response: We did not have a formal analysis protocol for the present study. This has been clarified in the main text (see page 7, line 137).

**C9: "Written informed consent" is mentioned twice early in the methods section, and once will suffice.**

Response: We have now removed the repetition of this information in the revised manuscript.

**C10: Throughout the text, please adapt reference call-outs to the following style: "... and their families [5,6]." (i.e., preceding punctuation and with no spaces in the square brackets).**

Response: We have now revised the referencing style throughout the revised manuscript.

**C11: In your reference list, please ensure that journal names are abbreviated consistently (e.g., "Lancet" and derivatives).**

Response: Done.

**C12: Please update reference 25, or supply the latest version with your revision.**

Response: Reference 25 has been updated.

**C13: Please remove the competing interest information from reference 32.**

Response: Done.

**C14: Please add a completed checklist for the most appropriate reporting guideline, which we suspect we will be STROBE, as a supplementary document, referred to in the methods section ("See S1_STROBE_Checklist" or similar). In the checklist, please refer to individual items by section (e.g., "Methods") and paragraph number rather than by line or page numbers, as the latter generally change in the event of publication.**

Response: A STROBE checklist for an observational cohort study has been included at the end of the supplementary appendices (see page 7, lines 137-139).

***Reviewer: 1***

**C1: I mostly confine my remarks to statistical aspects of this paper. One note is that it could use some editing for English usage, particularly the abstract.**

Response: The revised manuscript has been reviewed and edited by a native English speaking co-author.

**C2: On to statistics; the general method is fine, but I have some questions and issues to resolve before I can recommend publication. p. 2 - "Not alter the risks" .... not at all? Not significantly? Not in any clinically important way? Or what?**

Response: We have now clarified that the use of ventilation did not *statistically significantly* alter the risks of any outcomes in long-term solid fuel users (see page 2, line 51).

**C3: p. 6 - Categorizing continuous variables is a bad idea. If the data on age was collected this way then there is not much you can do (but it should be acknowledged) but if age was collected as "years" then leave it that way and look at a spline to examine nonlinearity**

Response: Thank you for the comment. We categorised the “duration of exposure (to solid fuel use)” because this information was collected in a semi-quantitative manner. Participants were only asked to recall the number of years lived in their three most recent residences and the corresponding fuel use in each of the residences. Individuals who had moved more frequently would therefore have less information on their historical household air pollution (HAP) exposure. The questionnaire also did not ask for the exact dates of moving in and out of each residence and participants reported this information in whole years. As a result, the difference in the recorded duration of exposure between participants may not accurately reflect exact number of years of exposure. Consequently, we believe the exposure metric is more appropriate to be ordinal instead of being continuous. We have explained our approach in more detail in our previous study (which had been cited when describing the exposure assessment methods), and we have now elaborated this explicitly in the revised manuscript (see page 8, lines 153-157). We have also acknowledged the lack of actual continuous exposure measurements as one of the key limitations of the present study (see page 20, lines 451-453).

**C4: p. 7 - Please give some details of how "direct standardization" was done.**

Response: We have now added further details about the direct standardisation method used in the supplementary appendices (see the revised Supplementary Methods). Below please find the corresponding text:

*“Direct standardisation was used to obtain age-, sex-, and study area-adjusted percentages or means of baseline characteristics to be compared across long-term cooking fuel exposure categories. Specifically, the original CKB study population was considered as the reference population, structured by 200 strata created from age groups (ten 5-year strata), sex, and ten study areas; then, adjusted percentages or means within each cooking fuel exposure group were estimated as* $\frac{\sum_{i} p_{i} \times\omega_{i}}{\sum_{i} \omega_{i}}$*, where* $p_{i}$ *are the raw percentages or means in the i^th^ stratum and* $\omega_{i}$ *are the weights (i.e. raw number of participant in the same stratum). Adjusted disease incidence rates were computed using the same approach, with adjusted rate estimated as* $\frac{\sum_{i} r_{i} \times\omega_{i}}{\sum_{i} \omega_{i}}$*, where* $r_{i}$*are the incidence rates in the i^th^ stratum and* $\omega_{i}$ *are the person-years at risk for each strata in the reference CKB population).”*

**C6: Tables - I commend the authors for leaving out p values. They really wouldn't add much.**

Response: We appreciate the reviewer’s positive comment.

***Reviewer: 2***

**C1: It is possible that UV exposure or outdoor air pollution has influenced the results. What do you think of the impact on these factors? Do you think the current analytic methods already has made relevant adjustments?**

Response: It is possible that UV or sunlight exposure is a potential risk factor for cataract, but the current epidemiological evidence remains inconclusive [2-4]. UV or sunlight exposure is strongly affected by occupation and physical activity, and these have been adjusted for in our analyses. Moreover, all analyses were adjusted for study areas so the differences in UV or sunlight exposure at different latitudes have been controlled for.

Similarly, severe outdoor air pollution has been suggested to be linked to acute conjunctivitis and glaucoma in some previous studies, but the totality of evidence remains limited and inconclusive. Our study participants tended to reside in close proximity in each of the ten study areas, so their outdoor air pollution exposure (at best classified based on geographic data down to 1km x 1km grid resolution) would have been similar. The inter-regional variations in outdoor air pollution should have been adjusted for (by the study area variable) in the multivariable analysis.

While we believe it is unlikely that our results were due to effects of UV exposure and outdoor air pollution, we have expanded the discussion to note the possibility of residual confounding from these or other related factors (e.g. agricultural dust exposure) as one of the limitations (see page 21, lines 479-490).

**C2: Why is the OR of 'never cook' as high as solid to clean? Shouldn't it be similar to control theoretically? Please add to discussion.**

Response: Thank you for the question. In households using solid fuels (primarily for cooking or heating), individuals who do not cook are also exposed to household air pollution (HAP) from the smoke generated. For this reason, in many studies HAP exposure has been classified based solely on the household fuel type (or stove type as proxy). Assuming those who did not cook to have zero exposure is therefore not realistic or appropriate. In the present study, household fuel type was only collected from participants who reported cooking regularly. As commonly seen in China, those participants who cooked were mostly women, and differed in many other ways from those who did not cook (e.g. age, occupation, smoking status). Direct comparison of those who cooked and those who did not was therefore also not appropriate as it would be subject to confounding. Hence, we focused on the exposure variation across regular-cooks who used different types of fuels. Overall, we observed similar risks among non-cooking individuals compared to solid fuel users, but we would like to avoid over-interpreting such findings due to the lack of exposure information. We have clarified this in the discussion section as suggested (see page 20, lines 453-457).

**C3: Dry eye disease is the most common ocular disease most likely to be affected by household air pollution. Is dry eye disease included in a conjunctival disorder or DSCIC in the current analysis?**

Response: When planning the present study, we selected disease categories with at least 1000 cases recorded during the follow-up period. After a 10-year follow-up, only 500 cases of potential dry eye disease (44 among clean fuel users) have been captured, half the minimum number to be included in the analysis. As this is an ongoing prospective study, further investigation can be conducted when more cases are available with even longer follow-up.

**C4: Retinal disease (macular degeneration or retinal vein occlusion) is one of the prevalent ocular disease. Why don't you include this?**

Response: There were only ~400 cases of retinal vascular occlusion and ~300 macular degeneration in the whole cohort, so they were not analysed as per the rationale explained in our response to C3 above. We have clarified this criterion in the methods section (see page 9, lines 178-179).

**C5: People who cook on solid fuels would be exposed to relatively strong firepower compared to clean fuels. Heavy heat exposure has the potential to exacerbate several eye diseases, including cataracts. What do you think it is possible that strong heat exposure from solid fuels, not HAP, could have resulted in this?**

Response: Thank you for the comment. Strong heat exposure is indeed a potential risk factor for some eye diseases such as cataract, but relevant epidemiological evidence relating to domestic solid fuel use is scarce [5,6]. Furthermore, the typical solid fuel stoves in China have confined fuel chamber(s) at knee-high level and both the chambers and fire pit/ stove top are usually insulated (e.g. with bricks or concrete) or covered by cooking utensils (see Figure 1 below). Therefore, we believe that it is unlikely that solid fuel users’ eyes are exposed to significantly more heat than clean fuel users in our study population (predominantly using gas, which burns at markedly higher temperature than solid fuels). Consistently, stove temperature data (recorded by a temperature logger placed on top of the stove) from previous HAP studies showed little evidence of strong heat emissions from solid fuel combustion [7]. Nevertheless, we have now included a discussion of heat exposure as a potential confounder (see page 21, lines 486-488).

**Figure 1: Typical stove types found in participants’ residence: coal briquette stove (left) and wood-fire stove (right)**

**C6: Disorders of sclera, cornea, iris and ciliary body (DSCIC) is a wide range of diseases category and it has diverse heterogenous pathophysiology respectively, so I think these results are not helpful clinically or academically. It would be better to exclude the DSCIC part from the results.**

Response: We agree that DSCIC is predominantly inflammation of one or several anterior structures of the eye (e.g. cornea, iris, ciliary body) and represents many heterogeneous conditions, and that one disease is a potential risk factor for the others [8]. Our work on DSCIC was primarily exploratory as research into specific eye diseases (other than cataract) in the HAP literature has been lacking. Our *a priori* hypothesis on the potential linkage between HAP and eye diseases was based on several common mechanisms (e.g. impaired immunity in the tear film, oxidative stress). We have further emphasised the exploratory and hypothesis-generating nature of the analysis on DSCIC (see page 17, lines 372-373, 376-379, 385-388).

***Reviewer: 3***

**C1: Abstract - clarify the reference for solid fuels in methods and findings.**

Response: We have clarified that accordingly (see page 2, lines 39-40, 44-45).

**C2: Page 7: Justify these exclusions further. Why not include these groups?**

Response: We have added explicit explanation for the exclusion criteria (see page 9, lines 183-190). Below please find the revised text:

*The present study excluded individuals who had missing data on body mass index (BMI; n=2); those who provided unreliable residential history (indicated by a difference between age and length of recall period >1 year; n=2189), those who reported using unspecified fuels at any residence (n=3342), and those who had ever switched from clean to solid fuels (n=15,150), because they are indicators of having potentially unreliable or unclear exposure profiles; and those who had cooked previously but stopped at baseline (n = 19,669), because their decision to stop cooking may be related to the disease outcomes of interest, thus risking reverse causation bias.*

**C3: Page 7: Provide reference for statement about bias in conventional survival analysis. Explain this statement.**

Response: We have clarified our statement about the risk of bias from delayed diagnosis of slowly developing eye diseases such as cataract, and how conventional survival analysis examining time-to-event may be particularly sensitive to such bias, when compared to logistic regression models that ignore time (see page 10, lines 197-206 and the new Supplementary Figure 1). Below please find the revised text and corresponding figure:

*“…Delays in diagnosis or treatment are particularly common for slowly progressing, non-lethal eye diseases such as cataract, especially in rural areas and among those at a lower socioeconomic status (SES)[9], who are more likely to be solid fuel users. Since conventional survival analysis examines time-to-event, the corresponding relative risk estimates would be more sensitive to biases that arise from the disproportionately longer delays in time-to-event among solid fuel users compared to clean fuel users (see* ***Supplementary Figure 1*** *for further explanation).”*

# *Supplementary Figure 1. Graphical illustration of potential bias from the disproportionately delayed treatment or diagnosis in solid fuel users*

Baseline

Diagnosis

Detectable cataract

Diagnosis

**Clean fuel users tend to have higher SES and live in urban areas**

**Solid fuel users tend to have lower SES and live in rural areas**

**Observed time-to-event (1)**

**Observed time-to-event (2)**

**Actual time-to-event**

*In this hypothetical scenario, assuming clean fuel and solid fuel users developed clinically detectable cataract at the same rate (i.e. no differences in risk as per the null hypothesis), since clean fuel users tend to have higher socioeconomic status (SES) and live in urban areas compared with solid fuel users, they have a higher chance of getting a timely diagnosis or treatment for their eye diseases such as cataract because of better access to healthcare [9]. The disproportionately longer delays in solid fuel users would generate misleading results suggesting solid fuel users have a lower rate (due to longer event-free period) of cataract than clean fuel users in conventional survival analysis. In contrast, logistic regression only examines event counts and is not affected by the timing of the events.*

**C4: Page 7: further justification is needed for confounders (e.g., a DAG or additional information).**

Response: We have added justification for the selection of confounders (see page 10, lines 209-213). Below please find the revised text:

*“…Confounder selection was based on a systematic literature search conducted on all risk factors of cataract and a standard modelling approach with covariates that contribute significantly to improve the log-likelihood ratio statistics included in the final models. Several key covariates (e.g. study area, cookstove ventilation, length of recall period) specific to the present study were included based on a priori knowledge.”*

**C5: Page 7: what method was used to assess interaction? An interaction term or stratification? Clarify. Be clear in the text that only multiplicative interaction was assessed; incorporate this fact into the results interpretation (any information then about additive interaction?)**

Response: We have only assessed multiplicative interaction (which is additive on a log-scale) but not additive interaction (on a linear scale), because our primary interest was to assess whether there was effect modification on the log-scale (as odds ratio is on the log-scale). We included an interaction term between cooking fuel and sex and cooking fuel use and smoking status in two separate regression models. These models were compared with those without the interaction term using a likelihood-ratio test statistic and the χ^2^ and p values of the interaction terms were also evaluated for statistical evidence of interaction. We have now clarified this in the methods, results and discussion sections (see page 11, lines 218-221; page 13, lines 269-276; page 16, lines 348-351)

We have also estimated the relative excess risk due to interaction (RERI) for conjunctiva disorders, cataract, and DSCIC (but not glaucoma, given the consistently null association observed) by fitting linear odds ratio models as described by Richardson & Kaufman[10] (see Table 1 below). The results are broadly consistent with those found for the multiplicative interactions, with evidence suggesting that solid fuel use for cooking is associated with significantly higher risk of cataract in women and never-smokers (who are predominantly women) than men and smokers, respectively. The RERI for DSCIC was marginally significant between solid fuel use and smoking, but this could be a chance finding (noting the wide confidence interval) and should be interpreted cautiously. Since the investigation of additive interactions were not part of our key research questions and the results do not offer additional insight, we prefer not to include this information in the revised manuscript.

| **Table 1. Relative excess risks of selected eye diseases due to interaction between solid fuel use for cooking and sex or smoking** | | |
| --- | --- | --- |
|  | **RERI (95% CI)** | |
|  | **Solid fuel + sex** | **Solid fuel + smoking** |
| Conjunctiva disorders | 0.14 (-0.03, 0.32) | 0.15 (-0.50, 0.36) |
| Cataract | 0.37 (0.25, 0.48) | 0.26 (0.17, 0.34) |
| DSCIC | 0.24 (-0.05, 0.52) | 0.35 (0.05, 0.64) |
| RERI: relative excess risk due to interaction; DSCIC: disorders of sclera, cornea, iris, and ciliary body. | | |

**C6: For interaction results it is not sufficient to just give p-value>0.05. Provide the p-value for each analysis. There actually seems to be a suggestion of interaction for cataracts by smoking status.**

Response: We thank the reviewer for noticing this. This was an error in the original version of the manuscript. There was indeed statistical evidence of interaction in this case. We have modified the results and discussion accordingly, and we have added the exact p-values for all other interactions investigated (page 13, lines 269-276; page 16, lines 348-351).

**C7: Was there any loss to follow up in the cohort? Need to report.**

Response: Thank you for the question. Between the baseline survey and January 1 2017, a total of 4,781 (0.9%) participants were lost to follow-up and censored in the analyses. We have now clarified this in the revised methods section (see page 9, lines 173-175).

**C8: A primary weakness of this study is the exposure assessment. Self-reported primary fuel use is a crude assessment of exposure. On Page 16 this limitation needs further discussion - including the potential impact on the observed results.**

Response: We have now discussed that limitation further (see pages 20, lines 445-457). Below please find the revised text:

*“…First, despite the enhancement in exposure assessment (combining personal cooking frequency and primary fuel type), it was not feasible to collect objectively measured HAP exposure data in the entire cohort and we had no information on household fuel use in never-regular cooks. It is possible that historical or concurrent exposure to HAP from secondary or neighbourhood fuels have elevated the background risk of eye disease in primary clean fuel users, and this could have diluted the associations examined. The lack of objective HAP data also prevented us from directly assessing the shape of the dose-response relationships of interest, although the findings on duration of exposure have offered some insight.”*

**C9: A second important limitation is residual confounding. This potential limitation needs to be discussed further, including potential impact on the results. How likely is residual confounding? By sunlight? By agricultural exposures? By smoking (due to mismeasured smoking status)?**

Response: Similar comments have been noted by Reviewer 2 (see our detailed response to Reviewer 2 C1 and C5). We have acknowledged residual confounding is an important limitation in the discussion section. (see page 21, lines 479-490). Below please find the revised text:

*“…Fifth, despite the extensive adjustment for a range of potential confounders, residual confounding from SES or smoking (due to reporting bias) or unmeasured confounders (e.g. sunlight, occupational dust, or ambient air pollution exposure) may still exist. For example, it is possible that individuals who used solid fuels, who were more likely to be agricultural workers in CKB, were exposed to more dust particles and sunlight, which are plausible risk factors for the eye diseases examined [11,12], and the associations may be overestimated due to residual confounding. Heat exposure related to cooking is another potential confounder for cataract, but there is no strong evidence to support that solid fuel users’ eyes were exposed to significantly higher radiation heat than clean fuel users (mostly using gas) [7]. Nonetheless, given the relatively small ORs observed and the large sample size, caution is required in the interpretation of these results due to residual confounding.”*

**C10: Provide additional details about ethics approvals.**

Response: Additional information about ethics approvals has been added (see page 7, lines 132-136).

**C11: Table 1: provide both N and %**

Response: Table 1 presents age-, sex-, and study area-adjusted percentages in order to allow meaningful (less confounded) comparison of baseline characteristics across different fuel use categories, so the raw number of participants in each cell would not correspond to the adjusted percentages and could be misleading. This is our group’s standard practice as shown in our previous publications [13-15] and we hope the reviewers and editors can appreciate its value.

**C12: The label "Never-regular cook" is not clear; be sure to clarify in Figures and tables.**

Response: We have now added footnote to clarify the meaning of “never-regular cook” in the relevant tables and figures.

***Reviewer 4:***

**C1. The manuscript purports to be of a prospective cohort study, but it has the characteristics of a cross-sectional study that has been followed up around a decade later, not necessarily with that original intent. Typically a well-performed prospective cohort study tracks participants, recollects data periodically, and ceases person-time contributions when a person is lost to follow-up (e.g., moves out of the study area) or dies. However, I see no evidence of tracking participant vital status and the hazard ratios and odds ratios appear to be based on the assumption of full cohort survival to the end of follow-up. This is likely to bias effect estimates towards the null. Adding to the impression of a cross-sectional study that has been followed up, is that all exposure and covariate data seems to have been collected at baseline and I see no evidence that participants were recontacted and data recollected. Use of baseline data limits the applicability of some of the covariates shown in Table 1, since they may have changed over the follow-up period.**

Response: We apologise for the ambiguity about the study design, particularly the follow-up methods and censoring criteria, which have now been clarified in the revised manuscript (see page 9, lines 169-176).

For clarification, the China Kadoorie Biobank (CKB) is a prospective cohort study with very broad research objectives, including those addressed by the present study [1]. Participants were surveyed at baseline during 2004-08 and have been followed continuously ever since for disease events and mortality via record linkage to death and disease registries and health insurance databases. The present study is an investigation of the association of HAP with incident eye disease events after 10-years of follow-up. It was infeasible to resurvey the entire cohort of 0.5 million participants, but periodic resurveys have been undertaken in a random subset (~5%) of the cohort every 4-5 years, in order to collect repeated measurements and additional data for enhancement. Further information about the cohort can be found in the study website: <https://www.ckbiobank.org>.

CKB has generated a substantial amount of research outputs that utilised a similar cohort study approach (prospectively ascertained outcomes in relation to exposure recorded at baseline) [13-20], although in the present report we chose to use logistic regression (instead of Cox regression) in the light of the risk of bias from delayed diagnosis of cataract (as explained in our response to Reviewer 3 C3). In fact, the use of logistic regression in longitudinal studies is common and, in some circumstances, has advantages over survival analysis [21,22].

**C2. A substantial limitation is that it appears that persons with aphakia or pseudoaphakia have not been excluded. A prospective cohort study with the prior intention of studying cataract would do that.**

Response: Although CKB was not set up specifically to study eye disease, by design, we excluded individuals with any major physical or mental disability which could affect their ability to complete the baseline data collection procedures. It is possible that some older participants would have developed cataract and undertaken operations to remove their cloudy lens, thus no longer at risk of cataract. Since older participants were more likely to be solid fuel users, the inclusion of individuals with aphakia, pseudoaphakia, or cataract could have diluted the associations. In an attempt to address this and the possible reverse causation from other subclinical or pre-existing eye conditions, we conducted a series of sensitivity analyses by excluding the first three years of follow-up, participants reporting poor health, and those aged ≥65 years at baseline (which should capture most of the individuals with a prior history of cataract), and found no material changes in the associations of interest (see Supplementary Table 3b). Overall, it is unlikely that prior cases of aphakia, pseudoaphakia or cataract have influenced the main findings. We have clarified this selection criteria in the methods section of the revised manuscript (see page 7, lines 124-125). The lack of information on prevalent eye conditions has been discussed as a limitation as well (see page 20, lines 459-463).

**C3. In the Introduction and the discussion, other study types are criticized on various grounds, including use of cross-sectional designs and use of household fuel types. These are essentially issues also with the present publication. The fact that it involved a 10-year follow-up, does not obviate the fact that it has essentially the limitations of a cross-sectional study. In fact, the limitations may be more severe: a cross-sectional study of eye diseases would typically submit every participant to clinical eye examination, but this study has relied on reporting to health insurance databases. There are two major consequences of this: (1) lack of consistent case-definitions (for example, what level of lens opacity becomes a cataract?): (2) likely underestimates of the true prevalence (or incidence) of the eye diseases investigated. Again, a bias likely towards the null.**

Response: We appreciate the reviewer’s comments. We recognise that different study designs entail different limitations and have acknowledged the range of limitations in the presence study.

Despite the choice of the primary analysis method (for reasons explained in our response to Reviewer 3 C3), the disease events were ascertained prospectively through robust record linkages to well-established registries and health insurance databases, and the exposure data were collected prior to the record of the disease events of interest. Therefore, the present study was able to assess the prospective associations (but not necessarily time-to-event) of HAP with eye diseases.

Performing regular clinical eye examinations in a prospective cohort study would be ideal to identify eye diseases. However, this was not feasible in a large study such as ours involving over 500,000 participants from geographically diverse areas. Indeed, some previous studies have relied on secondary data reported by hospitals as did our study. We have acknowledged this disadvantage in our study and have further expanded the corresponding discussion section in the revised manuscript (see page 20, lines 459-463).

**C4. It is mentioned in the methods that eight types of subgroup analyses (referred to as “sensitivity analyses” were carried out, but I saw no sign of those in the results. At least, a table of such results would seem appropriate.**

Response: The results of the sensitivity analyses are presented in Supplementary Tables 3a, 3b and 4 and briefly highlighted on page 14, lines 293-296 in the revised manuscript. These sensitivity analyses found no material changes in the overall associations of interest.

**C5. There is frequent use of the term “excess risk” throughout the manuscript, when reference is actually to relative risk estimates. Excess risk is a measure of absolute risk.**

Response: We apologise for the confusion. We have now changed the term to “higher risk” or “elevated risk” where appropriate.

**C6. Since they do not appear to have real measures of person-time, I suggest that they drop the use of Cox regression relative risks (hazard ratios). Odds ratios seem appropriate in this study, as (irrespective of the follow-up) it is effectively a cross-sectional study.**

Response: As per our response for C1 above, we have clarified the details of the follow-up methods, which provided us with person-time data for all participants in the cohort study (see page 9, lines 169-181). The choice of logistic regression was based on the risk of bias in relation to time-to-event rather than the analyses being based on cross-sectional data (see our response to Reviewer 3 C3 for further details). The supplementary results generated from the Cox regression analysis were presented to illustrate the potential bias we planned to avoid (see results for cataract) and the consistent findings for other diseases provides reassurance regarding the appropriateness of examining ORs.

**C7. The final adjusted study odds ratios are quite small and could easily be accounted for by some unmeasured confounder, such as socio-economic factors (measures of income, education and occupation were apparently collected, but these do not necessarily account for all socio-economic differences). Although the confidence intervals exclude the null, the calculations are based on such large numbers of participants, that small ORs (easily accounted for by biases, including confounding) often appear statistically significant.**

Response: We have acknowledged the possibility of residual confounding, particularly from socio-economic factors and other unmeasured plausible confounders. We have also emphasised the need of cautious interpretation of our findings and further studies with better clinical ascertainment of eye diseases and potential confounders (see also our response to Reviewer 3 C9; main text pages 2-3, lines 51-55; page 21, lines 479-490).

**C8. I suggest that separate analyses be carried out for men and for women, as their exposures are likely to be quite different and mixing them together may obscure important results.**

Response: In the original manuscript, we presented sex-specific analyses as part of the subgroup analyses (see Supplementary Figure 3) and discussed the findings accordingly. Apart from cataract, there was no evidence of effect modification by sex on the relationship between fuel use exposure and eye disease risk. Unlike previous studies examining only household fuel or stove types, we classified exposure by personal cooking behaviour and focused on individuals who cooked regularly regardless of gender. Although limited evidence suggests that men cooked slightly less intensely and frequently than women even among regular cooks, we believe the overall ORs (combining men and women) still serve to highlight the importance of focussing on personal exposure (see page 15, lines 332-339).

**C9. I would prefer final results to be shown in tables, but if figures are to be retained, it would be better to label the ends of the confidence intervals with their magnitudes. I am not clear why the reference categories have confidence intervals. Also, the fact that in Figure 1, never-regular cooks have odds ratios similar to always solid fuels deserves some comment.**

Response: We have added the exact numbers for the group-specific CIs presented in the figure legends of all main figures.

The reference categories have confidence intervals because we have calculated group-specific CIs using the variance of the log odds in each category (with the point estimate remaining unaltered). This is routinely done in our analyses and widely used in similar studies from this and other groups [13-15,23-25]. Compared to the conventional way of presenting CIs only for the comparison groups (with reference to one single category), this method allows comparisons of the relative risk estimates of any two categories of exposure [23]. This is particularly important in our study examining disease risks across multiple categories of exposure of interest, as we are not only interested in comparing the disease risk of all categories to one single reference category (i.e. clean fuel users). We have clarified this further in the main text and a reference to a methodology paper has been included (see page 11, lines 235-241).

We have also added more explicit comments about the possible reason why never-regular cooks have similar odds ratio to long-term solid fuel users (see our response to Reviewer 2 C2; main text page 20, lines 453-455).

**C10. In the first sentence of the second paragraph of the discussion, the authors appear to be criticizing other studies for focusing on “age-related cataract”, as if this is a different type of cataract to the type they are focusing on. I think this impression was not intentional, but if they are going to use this term, they should use it in a more general sense at the beginning of the article, as all cataract is essentially age-related.**

Response: We apologise for the unintended impression. The term “age-related cataract” is a well-defined clinical outcome under the International Classification of Diseases, 10^th^ Revision. It is defined as cataracts occurring in people >50 years of age, in the absence of known mechanical, chemical, or radiation trauma [26]. A range of cataracts (e.g. diabetic cataract, radiation induced cataract, traumatic cataract and drug-induced cataract) are not considered as “age-related cataract”, even though people may acquire it during late adulthood [26]. This term has been widely used in clinical settings (<https://www.nhs.uk/conditions/cataracts/>), academic literature on eye disease [27], and the previous literature on the topic of interest (analysed as the primary outcome in 6 out of 10 relevant studies identified via a systematic literature search). While in several studies the outcome assessment method was ambiguous, we had no intention to criticise this choice of the outcome, but merely to highlight the fact that most previous studies focused on this type of cataract, which is also the predominant type in our study. We have revised the wordings and supplied additional information to clarify the issue (see page 14, lines 310-313).

**C11. Towards the end of the discussion, the authors refer to “a few limitations”, almost as an afterthought. Some of them correspond with my criticisms above, but, in my view, the study limitations are sufficiently major that this part of the discussion should be expanded and emphasized in importance."**

Response: We apologise for the unintended impression. We have expanded the discussion on the key limitations substantially and emphasised their importance as suggested (see page 19-21, lines 444-490).

**References**

1.	Chen Z, Chen J, Collins R, Guo Y, Peto R, Wu F, et al. China Kadoorie Biobank of 0.5 million people: survey methods, baseline characteristics and long-term follow-up. Int J Epidemiol. 2011;40(6):1652-66.

2.	Klein BEK, Cruickshanks KJ, Klein R. Leisure time, sunlight exposure and cataracts. Documenta Ophthalmologica. 1995;88(3):295-305.

3.	Bochow TW, West SK, Azar A, Munoz B, Sommer A, Taylor HR. Ultraviolet light exposure and risk of posterior subcapsular cataracts. Arch Ophthalmol. 1989;107(3):369-72.

4.	Roberts JE. Ultraviolet radiation as a risk factor for cataract and macular degeneration. Eye Contact Lens. 2011;37(4):246-9.

5.	Sharon N, Bar-Yoseph PZ, Bormusov E, Dovrat A. Simulation of heat exposure and damage to the eye lens in a neighborhood bakery. Exp Eye Res. 2008;87(1):49-55.

6.	Nandi SK, Rankenberg J, Glomb MA, Nagaraj RH. Transient elevation of temperature promotes cross-linking of α-crystallin-client proteins through formation of advanced glycation endproducts: A potential role in presbyopia and cataracts. Biochem Biophys Res Commun. 2020.

7.	Ruiz-Mercado I, Canuz E, Smith KR. Temperature dataloggers as stove use monitors (SUMs): Field methods and signal analysis. Biomass Bioenergy. 2012;47:459-68.

8.	Nien CW, Lee CY, Chao SC, Hsu HJ, Huang JY, Yeh CB, et al. Effect of Uveitis on the Development of Keratopathy: A Population-Based Cohort Study. Invest Ophthalmol Vis Sci. 2018;59(12):5053-9.

9.	World Health Organisation. World report on vision. Switzerland: World Health Organization; 2019.

10.	Richardson DB, Kaufman JS. Estimation of the Relative Excess Risk Due to Interaction and Associated Confidence Bounds. Am J Epidemiol. 2009;169(6):756-60.

11.	Hurst TS, Dosman JA. Characterization of health effects of grain dust exposures. Am J Ind Med. 1990;17(1):27-32.

12.	Delavar A, Freedman DM, Velazquez-Kronen R, Little MP, Kitahara CM, Alexander BH, et al. Ultraviolet radiation and incidence of cataracts in a nationwide US cohort. Ophthalmic Epidemiol. 2018;25(5-6):403-11.

13.	Du H, Li L, Bennett D, Guo Y, Turnbull I, Yang L, et al. Fresh fruit consumption in relation to incident diabetes and diabetic vascular complications: A 7-y prospective study of 0.5 million Chinese adults. PLoS Med. 2017;14(4):e1002279.

14.	Chan KH, Kurmi O, Bennett D, Yang L, Chen Y, Tan Y, et al. Solid fuel use and risks of respiratory diseases: a cohort study of 280,000 Chinese never-smokers. Am J Respir Crit Care Med. 2019;199(3):352-61.

15.	Du H, Li L, Bennett D, Guo Y, Key TJ, Bian Z, et al. Fresh fruit consumption and major cardiovascular disease in China. N Engl J Med. 2016;374(14):1332-43.

16.	Chen ZM, Peto R, Iona A, Guo Y, Chen YP, Bian Z, et al. Emerging tobacco-related cancer risks in China: A nationwide, prospective study of 0.5 million adults. Cancer. 2015;121 Suppl 17:3097-106.

17.	Bragg F, Li L, Bennett D, Guo Y, Lewington S, Bian Z, et al. Association of Random Plasma Glucose Levels With the Risk for Cardiovascular Disease Among Chinese Adults Without Known Diabetes. JAMA Cardiol. 2016;1(7):813-23.

18.	Bragg F, Li L, Yang L, Guo Y, Chen Y, Bian Z, et al. Risks and Population Burden of Cardiovascular Diseases Associated with Diabetes in China: A Prospective Study of 0.5 Million Adults. PLoS Med. 2016;13(7):e1002026.

19.	Bragg F, Holmes MV, Iona A, Guo Y, Du H, Chen Y, et al. Association between diabetes and cause-specific mortality in rural and urban areas of China. JAMA. 2017;317(3):280-9.

20.	Yu K, Qiu G, Chan KH, Lam KH, Kurmi OP, Bennett DA, et al. Association of Solid Fuel Use With Risk of Cardiovascular and All-Cause Mortality in Rural China. JAMA. 2018;319(13):1351-61.

21.	Annesi I, Moreau T, Lellouch J. Efficiency of the logistic regression and Cox proportional hazards models in longitudinal studies. Stat Med. 1989;8(12):1515-21.

22.	Staley JR, Jones E, Kaptoge S, Butterworth AS, Sweeting MJ, Wood AM, et al. A comparison of Cox and logistic regression for use in genome-wide association studies of cohort and case-cohort design. Eur J Hum Genet. 2017;25(7):854-62.

23.	Plummer M. Improved estimates of floating absolute risk. Stat Med. 2004;23:93-104.

24.	Chen Z, Peto R, Zhou M, Iona A, Smith M, Yang L, et al. Contrasting male and female trends in tobacco-attributed mortality in China: evidence from successive nationwide prospective cohort studies. Lancet. 2015;386(10002):1447-56.

25.	Di Angelantonio E, Chowdhury R, Sarwar N, Aspelund T, Danesh J, Gudnason V. Chronic kidney disease and risk of major cardiovascular disease and non-vascular mortality: prospective population based cohort study. BMJ. 2010;341:c4986.

26.	Allen D. Cataract. BMJ Clin Evid. 2011:0708.

27.	Asbell PA, Dualan I, Mindel J, Brocks D, Ahmad M, Epstein S. Age-related cataract. Lancet. 2005;365(9459):599-609.
