# Supplementary material for: Long-term solid fuel use and risks of major eye diseases in China: A population-based cohort study of 486,532 adults
Source: PLoS Med. 2021 Jul 29;18(7):e1003716. doi: 10.1371/journal.pmed.1003716 (PMC8321372; doi:10.1371/journal.pmed.1003716)
Supplement: Attachment — Submitted filename: PMEDICINE-D-20-04664R2_Response_21JAN2021.docx [file pmed.1003716.s006.docx]

21 Jan 2021

Dr Richard Turner, PhD
Senior Editor
PLOS Medicine

Dear Dr Turner,

**Re: PMEDICINE-D-20-04664R2**

Thank you again for the opportunity to revise the above manuscript for further consideration for publication in the PLOS Medicine. We have revised the manuscript in the light of the constructive feedback from the editors and reviewers. Attached are our point-by-point responses to the comments. Verbatim comments are shown below in bold, and our responses are shown in non-bold text, with page and line numbers indicating changes in the revised manuscript.

Huaidong Du
Senior Research Fellow

Nuffield Department of Population Health
University of Oxford
United Kingdom

Kin Bong Hubert Lam
Associate Professor

Nuffield Department of Population Health
University of Oxford
United Kingdom

Zhengming Chen

Professor of Epidemiology

Nuffield Department of Population Health

University of Oxford

United Kingdom

**Response to comments**

Manuscript no.: PMEDICINE-D-20-04664R2

***Editors:***

**C1: Please revisit the wording at line 118 and any other instances, noting the further comments from one referee - we continue to view the study as a retrospective analysis of prospectively gathered data and ask you to adapt the language used to reflect the study design.**

Response: We appreciate the editors and reviewers’ views, and we have now revised the manuscript to avoid describing the present study as a “prospective cohort” as suggested.

**C2: Where appropriate, please substitute "p<0.001" for "p<0.0001" throughout the ms.**

Response: Done.

**C3: Please substitute "sex" for "gender" throughout, again where appropriate.**

Response: Done.

***Reviewer: 2***

**C1: I cannot find the evidence that there is no difference of heat exposure between solid and clean fuel in your reference. Solid fuel can expose a lot of heat to the eyes during the first ignition process or fire boosting.**

Response: We have now further expanded the discussion about the potential residual confounding from heat exposure associated with solid fuel use for cooking (see page 22, lines 501-506). To the best of our knowledge, no previous studies have measured temperature exposure of the eyes in individuals using solid or clean fuels for domestic cooking, so we cited studies that had measured temperature around solid fuel cookstoves in our response. We have now highlighted the need for further studies to measure heat exposure alongside household air pollution to tease out the associations of these two exposures with eye disease.

**C2: In addition, dry eye disease is the most prevalent disease in the world, and the known prevalence in the world is about 15-20%.**

Response: The CKB was designed as a general cohort study, and the record linkage method employed is best suited to capture major disease events requiring treatment in hospitals or health insurance reimbursement. Given most cases of dry eye disease are relatively mild (e.g., requiring only behavioural intervention or over-the-counter eyedrops), it was not surprising to see a relatively small number of events in CKB (<500 in the whole cohort, ~40 in clean fuels reference group), which would lead to severely underpowered statistical analyses. Therefore, we have not included these events in the present manuscript. However, we have further clarified the nature of the follow-up method, the associated limitations, and the criteria of selecting disease outcomes for this study (see page 9, lines 168-172 and 178-180; page 21, lines 483-492).

**C3: UV or sunlight exposure should also be regarded as an independent risk factor in the ocular disease.**

Response: We agree with the reviewer that UV or sunlight exposure is a potential confounder in the present study. As we did not collect direct or objective data on UV or sunlight exposure for individual participants (which are also rarely available in previous epidemiological studies), we have attempted to account for this by adjusting for several key proxy indicators, including (i) occupation (a major determinant of sunlight exposure in our study population, with agricultural workers likely exposed to markedly higher levels); (ii) physical activity levels (associated with time spent outdoors), and; (iii) study area (a proxy for latitude, a major determinant of UV exposure). These adjustments had little impact on the odds ratios (ORs) of interest. However, we still acknowledge that residual confounding is likely to be remain after these imperfect adjustments (see pages 21-22, lines 492-501).

We hope our latest response to C1-C3 and corresponding changes in the manuscript, especially those related to discussion on study limitations have now addressed the reviewer’s concerns.

***Reviewer: 3***

**C1: 1. I see that the Editors requested p-values along with 95% CIs; I don't agree with this suggestion, but note that the authors did this to comply with the editors request. Please note the various recommendations throughout the statistical and epidemiologic literature to decrease emphasis on p-values. Perhaps this can be modified in the final version. Overall there is too much emphasis on statistical significance in the current version. In this regard, the sentence in the abstract about ventilation could be modified to state clearly that the ORs are similar regardless of ventilation, rather than the focus on the statistical significance (the statistical significance supports this result, it's not the primary result).**

Response: We agree with the reviewer that statistical significance should not be over-emphasised and that focus should be on the point estimates and their associated confidence intervals. Considering the comments from the editors and the previous Reviewer 3 together, we have now revised the manuscript to keep only the key p-values (e.g., in the abstract, where more detailed results are lacking; and for tests for interaction enquired by the Reviewer) and focus on ORs, confidence intervals and clinical significance in the main text.

**C2: I suggest not using the abbreviation HAP. Although common in this field, it also means hazardous air pollutants.**

Response: We have now removed the abbreviation HAP.

**C3: I still have a concern about approach to confounding. There is no statistical test for confounding, despite what you sometimes see in the literature. The statistical tests are evaluating other aspects, not confounding. Once confounders have been identified (and DAGs or other methods used to make sure you are not adjusting for mediators or colliders) there is value perhaps to reducing the number of variables in the model (e.g., if removing them does not change the ORs in a meaningful way). There was also no reference cited for the approach. Please rely here on the epidemiologic approach to confounding, and not a statistical test.**

Response: We apologise for the ambiguity. We have now clarified the approach further and supplied a relevant reference (see pages 10, lines 210-217). We adopted an approach to confounding described by VanderWeele [1], whereby we identified the potential confounders based on prior knowledge about the potential linkages between the exposure, outcomes, and confounders from the existing literature (including our previous studies on household air pollution). The VanderWeele paper also highlights that the statistical approach we have utilised (i.e., sequential adjustment with likelihood ratio tests) is more appropriate than the epidemiological approach of using the change in estimates to assess confounding, as the latter may miss some confounders due to the non-collapsibility of the OR.

***Reviewer 4:***

**C1. The main one is that the study purports to be superior to previous studies because it is "prospective". In fact, a true prospective study would have carried out at least baseline eye examinations of the whole cohort and excluded those already with cataract, etc., from the multivariate models. It would then be possible to ascertain which of the diagnosed conditions prospectively occurred during the period of follow-up (preferably with periodic eye examinations of the whole cohort). The fact that baseline eye examinations did not occur means that the study more closely resembles a retrospective cohort study, in which a cohort is identified in the past and tracked into the future, using registries to identify diagnoses that have occurred since the start of the follow-up period. In such studies, there is always the possibility that the diseases of interest were present at the start of the follow-up period. The authors correctly identify the lack of baseline examinations as being a deficiency, but persist in calling it a prospective study (or at least implying that it is). Examples include:**

***	Abstract, line 31. "Little reliable prospective evidence exists…."**

***	Author summary, lines 68-69. "…but most previous studies used retrospectively collected data"**

***	Lines 86-87. "…this is the first investigation on the prospective relationship between long-term solid fuel use and risks of multiple common eye diseases."**

***	Lines 314-315 (in reference to previous studies): "Notably, all these studies were relatively small, cannot assess the temporality of association…"**

***	Line 323 (again, in reference to previous studies): "…they suffered from similar limitations, particularly the inability to assess temporality."**

***	Line 443. "The strengths of this study are the prospective follow-up…"**

**The authors reasonably assume, for SES reasons, that solid fuel users are likely to have a longer time to diagnosis than clean fuel users. This makes it even more essential that identification of baseline abnormalities and subsequent exclusions took place before claims of the superiority of their analysis can be made (examples above). They use the "sensitivity analyses" to argue that the lack of baseline examinations is not a problem, but this is a very indirect, weak and likely insensitive method of inference. The deficiency of uniform baseline (and subsequent) eye examinations is likely to impact logistic regression analysis as much as survival analysis (in contrast to their response to reviewer 3 and arguments on lines 201-208), and the bias is likely to be towards the null (assuming a HAP effect). The reason for this is that many solid fuel users may never be diagnosed within the follow up period of the study, or are certainly less likely to be diagnosed than higher SES clean fuel users.**

Response: We apologise for unintended impression. In the revised manuscript, we have clarified this in the main text to avoid confusion (page 2, line 30; page 4, lines 67-68, 72-73, 85-86; page 6, line 109-111; page 15, lines 318-327; pages 20-21, lines 464-492). We agree that a better prospective study on eye disease would satisfy the criteria suggested by the reviewer, but this was not feasible for a large-scale study of >500,000 participants. Our cohort was designed to follow-up a large number of participants for a wide range of exposures and diseases, and regretfully, some level of granularity had to be sacrificed (see Chen 2020 [2] for the detailed rationale). Apart from further highlighting the limitations in our study (see pages 20-21, lines 464-492), we have also emphasised the importance of developing better prospective studies of eye diseases along the lines described by the reviewer (see page 22, lines 517-522).

Our sensitivity analyses (e.g., resetting the baseline to 3-years later by removing all individuals who have developed any disease events or died during the first 3 years; or removing participants aged ≥65 years who would have accounted for most pre-existing cataract cases[3]) were undertaken to assess the impact of bias induced by pre-existing eye disease cases on the findings. We acknowledge that although these exclusions may reduce the impact, they will not eliminate the bias. This remains a key limitation of the study, particularly for the findings on cataract and glaucoma (but perhaps less so for other acute infection outcomes that can occur multiple times and independently), and this is acknowledged in the discussion section of the revised report (see page 21, lines 477-483).

**C2. My second concern is that the results and differences between males and females in terms of eye diseases is always of major interest and importance when comparing results between different studies, particularly with the HAP issue. I do not think this information should be buried in the supplementary material (Supplementary Figure 3). Also, all the supplementary figures should have the same information (i.e., confidence intervals) as the figures in the main paper. I would make a similar argument for the interaction with smoking (Supplementary Figure 2), which deserves to be elevated from the supplementary material.**

Response: We have now reformatted the figures and moved the two figures on subgroup analysis to the main text as suggested.

**C3. Why is BMI a covariate? What is the evidence that it may be a confounder—for all the eye diseases studied?**

Response: Adiposity is known to be associated with impaired immunity and elevated risks of infections [4], and is a strong risk factor for diabetes and insulin resistance, which are in turn major risk factors for eye diseases and impaired immunity [5,6]. Solid fuel users tend to have a lower socioeconomic status, and thus are more likely to have different weight profiles compared to the more affluent clean fuel users. Therefore, we adjusted for BMI as a proxy of the potential confounding effects of adiposity.

**References**

1.	VanderWeele TJ. Principles of confounder selection. Eur J Epidemiol. 2019;34(3):211-9.

2.	Chen Z. Chapter 2: Design, implementation, and managment of biobank studies. In: Chen Z, editor. Population biobank studies: a practical guide. Singapore: Spinger Nature Singapore; 2020.

3.	Song P, Wang H, Theodoratou E, Chan KY, Rudan I. The national and subnational prevalence of cataract and cataract blindness in China: a systematic review and meta-analysis. J Glob Health. 2018;8(1):010804.

4.	de Heredia FP, Gómez-Martínez S, Marcos A. Obesity, inflammation and the immune system. Proc Nutr Soc. 2012;71(2):332-8.

5.	Negi A, Vernon SA. An Overview of the Eye in Diabetes. J Royal Soc Med. 2003;96(6):266-72.

6.	Ansari AS, de Lusignan S, Arrowsmith B, Hinton W, Munro N, McGovern A. Association Between Diabetes, Level of Glycemic Control, and Eye Infection: A Cohort Study. Diabetes Care. 2017;40(3):e30-e1.
