# Supplementary material for: Long-term solid fuel use and risks of major eye diseases in China: A population-based cohort study of 486,532 adults
Source: PLoS Med. 2021 Jul 29;18(7):e1003716. doi: 10.1371/journal.pmed.1003716 (PMC8321372; doi:10.1371/journal.pmed.1003716)
Supplement: Attachment — Submitted filename: PMEDICINE-D-20-04664R4_Response.docx [file pmed.1003716.s008.docx]

28 June 2021

Dr Richard Turner, PhD
Senior Editor
PLOS Medicine

Dear Dr Turner,

**Re: PMEDICINE-D-20-04664R4**

Thank you again for the opportunity to revise the above manuscript for further consideration for publication in the PLOS Medicine. We are including our point-by-point responses to the requests from the Editors. We are also attaching our responses to the comments from the previous round of review for your convenience.

Huaidong Du
Senior Research Fellow

Nuffield Department of Population Health
University of Oxford
United Kingdom

Kin Bong Hubert Lam
Associate Professor

Nuffield Department of Population Health
University of Oxford
United Kingdom

Zhengming Chen

Professor of Epidemiology

Nuffield Department of Population Health

University of Oxford

United Kingdom

**Response to comments**

Manuscript no.: PMEDICINE-D-20-04664R4

**C1** **Please adapt the data statement (submission form) to explain briefly the nature of restrictions on access to data - e.g., access can only be granted to investigators for stated research purposes. Please remove information on data access from the end of the main text. Again, this information should appear only in the article metadata.**

Response: We have now removed the data access statement from the main text and provided that via the submission form as suggested.

**C2: In the abstract, please make that "cataracts" (line 38) and "cases of glaucoma" (line 39), or similar, and adapt the phrasing similarly throughout the ms.**

Response: Done.

**C3:** **At line 94 (author summary) please revisit the statement "... appeared to be considerably weaker.". It appears that a few words need to be added to state the conditions under which the association is weaker.**

Response: We have edited the sentence to indicate that the comparison was made to findings from previous studies, with the revised text as follows:

*“Our findings support a significant association between solid fuel use and cataracts, but the strength of association appeared to be considerably weaker compared to that observed in previous studies”*

**C4: Please remove the information on study funding from the "Acknowledgements" at the end of the main text Information on funding should appear only in the article metadata, via entries in the submission form.**

Response: Done.

**C5: Please add a sentence, say, early in the "Methods and findings" subsection of your abstract to summarize the factors adjusted for.**

Response: A sentence (page 2, lines 42-45) has now been added to summarize the factors adjusted in the main analyses as follows:

“…with adjustment for age at baseline, birth cohort, gender, study area, education, occupation, alcohol intake, smoking, environmental tobacco smoke, cookstove ventilation, heating fuel exposure, body mass index, prevalent diabetes, self-reported general health, and length of recall period.”

**C6: Please remove the names of the CKB group from the end of the main text, and list these in a supplementary file.**

Response: Done.

**C7: Please substitute "sex" for "gender" where appropriate throughout the ms, e.g., at line 42 in the abstract and in table 2.**

Response: We have performed the search function and have not been able to locate the word “sex” in the manuscript (PMEDICINE-D-20-04664R3), except when it appeared in the title of a reference that we cited.

**From the previous round of review (PMEDICINE-D-20-04664R3)**

***Editors:***

**C1: We suggest addressing referee 2's second point by mentioning this issue as a possible limitation in the Discussion section (main text).**

Response: Thank you for the suggestion. We have now further discussed the issue as a possible limitation in the main text (page 17, lines 396-402) as follows:

*“Despite the relatively large sample size, our study lacked the power to investigate the associations of solid fuel use with each of the specific DSCIC, which have heterogeneous pathophysiology and may not necessarily be subject to the same impact from household air pollution. In the absence of previous studies on household air pollution and DSCIC, our study has generated a new hypothesis that warrants further investigation on the association of solid fuel use with each of the specific DSCIC.”*

**C2: Please quote the actual number of participants in the title rather than "0.5 million", and again at line 74.**

Response: Done.

**C3: We suggest mentioning the China Kadoorie Biobank in the abstract.**

Response: Done.

**C4: Please quote the actual length of follow-up, as at line 259, in the abstract.**

Response: Done.

**C5: Please add a sentence, say, early in the "Methods and findings" subsection of your abstract to summarize the factors adjusted for.**

Response: A sentence (page 2, lines 42-45) has now been added to summarize the factors adjusted in the main analyses as follows:

“…with adjustment for age at baseline, birth cohort, gender, study area, education, occupation, alcohol intake, smoking, environmental tobacco smoke, cookstove ventilation, heating fuel exposure, body mass index, prevalent diabetes, self-reported general health, and length of recall period.”

**C6: At line 264, for example, please substitute "sex" for "gender" as appropriate.**

Response: We have now replaced the term “sex” with “gender” throughout the manuscript.

**C7: Please use the journal name abbreviation "PLoS ONE" in the reference list.**

Response: The reference number 45 has been corrected.

**C8: Please break the STROBE checklist out into a separate attached file, labelled "S1_STROBE_Checklist" or similar, and refer to it by this label in the Methods section.**

Response: Done.

***Reviewer #2:***

**C1: ) In the clinical aspects, conjunctival disorder, which you showed the major disorder related with solid fuel, are also relatively mild eye disease, similarly with dry eye disease. In addition, dry eye disease is more prevalent in East Asia (China, Korea, Japan, etc) In previous study using meta-analysis in China, pooled prevalence of dry eye disease in China was 17.0%. (Liu et al, J Ophthalmol. 2014;2014:748654.) In my opinion, if patients go to hospital due to conjunctival disorder frequently, patients also go to hospital due to dry eye disease similarly. If not, it is also possible that a patient with dry eye disease was incorrectly classified as a conjunctival disorder. In dry eye patients, conjunctival injections are very frequent. Therefore, I think this cohort data may not be reflect the real world situation adequately. Authors should emphasize strongly the possibility of underestimation or false classification of dry eye disease as a limitation.**

Response: We appreciate the reviewer’s comments and we have now discussed the possibility of underestimation or misclassification of dry eye disease as a limitation of the record linkage data we used in the present study (see page 21, lines 495-503).

“*The use of the national health insurance data also constrained the study to more severe eye disease events requiring treatment in hospitals or health insurance reimbursement (as opposed to simple over-the-counter drug treatment) and omitted other potentially prevalent conditions such as dry eye disease [1,2], which has been linked to solid fuel use in previous studies [3]. It is also possible that patients with mild dry eye disease were misclassified as having conjunctivitis because dry eye disease could be secondary to conjunctivitis and they usually share some common symptoms (e.g., redness, itchiness, stinging), especially given the general lack of objective or laboratory-confirmed diagnoses in China [2].*”

**C2: In my previous review, I recommended to exclude DSCIC part from the results. Disorders of sclera, cornea, iris and ciliary body (DSCIC) is a wide range of diseases category and it has diverse heterogenous pathophysiology respectively, so I think these results cannot be helpful clinically or academically, even if I review it again now. Please exclude the results of DSCIC.**

Response: As suggested by the Editors, we have kept the results on DSCIC but further highlighted the reviewer’s concern as a limitation of the study, and we call for further investigation on each the specific conditions future studies (see page 17, lines 396-402).

*“Despite the relatively large sample size, our study lacked the power to investigate the associations of solid fuel use with each of the specific DSCIC, which have heterogeneous pathophysiology and may not necessarily be subject to the same impact from household air pollution. In the absence of previous studies on household air pollution and DSCIC, our study has generated a new hypothesis that warrants further investigation on the association of solid fuel use with each of the specific DSCIC.”*

***Reviewer: 3***

**C1: 1. The authors have addressed my concerns. I continue to question BMI as a confounder given its potential role as a mediator; this potential role should at least be acknowledged.**

Response: Thank you for the feedback. Indeed some literature suggest that ambient air pollution may be associated with increased risks of metabolic dysfunction and thus obesity (which is associated with elevated risk of eye diseases such as cataract), but the overall evidence remains mixed [4]. There is even less evidence on the role of household air pollution from solid fuels, which is known to be markedly different from ambient air pollution. In our study, solid fuel use was associated with modestly lower BMI (-0.5kg/m^2^ compared to clean fuel use), which likely reflect the impact of poverty (a common cause of low BMI and solid fuel use) and oppose the aforementioned mediation mechanism hypothesis. Furthermore, removing BMI from our analytical models did not result in material change in our findings, again suggesting no mediating role of BMI in this study.

**References**

1.	Song P, Xia W, Wang M, Chang X, Wang J, Jin S, et al. Variations of dry eye disease prevalence by age, sex and geographic characteristics in China: a systematic review and meta-analysis. J Glob Health. 2018;8(2):020503.

2.	Liu N-n, Liu L, Li J, Sun Y-z. Prevalence of and Risk Factors for Dry Eye Symptom in Mainland China: A Systematic Review and Meta-Analysis. Journal of Ophthalmology. 2014;2014:748654.

3.	Łatka P, Nowakowska D, Nowomiejska K, Rejdak R. How air pollution affects the eyes — a review. Ophthalmology Journal. 2018;3(2):58-62.

4.	An R, Ji M, Yan H, Guan C. Impact of ambient air pollution on obesity: a systematic review. International Journal of Obesity. 2018;42(6):1112-26.
